# Dielectric metasurfaces for complete and independent control of the optical amplitude and phase

**DOI:** 10.1038/s41377-019-0201-7

**Published:** 2019-10-09

**Authors:** Adam C. Overvig, Sajan Shrestha, Stephanie C. Malek, Ming Lu, Aaron Stein, Changxi Zheng, Nanfang Yu

**Affiliations:** 1 Department of Applied Physics and Applied Mathematics, Columbia University, New York, NY 10027 USA; 2 Center for Functional Nanomaterials, Brookhaven National Laboratory, Upton, NY 11973 USA; 3 Department of Computer Science, Columbia University, New York, NY 10027 USA

**Keywords:** Metamaterials, Nanophotonics and plasmonics

## Abstract

Metasurfaces are optically thin metamaterials that promise complete control of the wavefront of light but are primarily used to control only the phase of light. Here, we present an approach, simple in concept and in practice, that uses meta-atoms with a varying degree of form birefringence and rotation angles to create high-efficiency dielectric metasurfaces that control both the optical amplitude and phase at one or two frequencies. This opens up applications in computer-generated holography, allowing faithful reproduction of both the phase and amplitude of a target holographic scene without the iterative algorithms required in phase-only holography. We demonstrate all-dielectric metasurface holograms with independent and complete control of the amplitude and phase at up to two optical frequencies simultaneously to generate two- and three-dimensional holographic objects. We show that phase-amplitude metasurfaces enable a few features not attainable in phase-only holography; these include creating artifact-free two-dimensional holographic images, encoding phase and amplitude profiles separately at the object plane, encoding intensity profiles at the metasurface and object planes separately, and controlling the surface textures of three-dimensional holographic objects.

## Metasurfaces: next generation holograms

Dielectric metasurfaces that can independently control the phase and amplitude of two wavelengths of light simultaneously are bringing new opportunities for computer-generated holography. Adam Overvig and coworkers from Columbia University and Brookhaven National Laboratory in the US have shown that such metasurfaces make it possible to control the surface texture of three-dimensional holographic objects, create artifact-free two-dimensional holographic images and support two-colour operation. The dielectric metasurface holograms are made by growing an 800–1000 nm thick layer of amorphous silicon onto a quartz wafer and then using electron-beam lithography and reactive ion etching to write holograms into the silicon layer. The team says that the fabrication process is CMOS compatible and the resulting metasurface holograms measure ~750x750 µm and operate in transmission mode with high efficiency.

## Introduction

Structuring materials for arbitrary control of an optical wavefront is a long sought-after capability, enabling any physically possible linear optical functionality. Four key properties of a light wave are the amplitude, phase, polarization, and optical impedance. The ability to tune these properties at specific frequencies with subwavelength spatial resolution is the goal and promise of a class of metamaterials known as “metasurfaces”, flat optical components composed of subwavelength structures with tailored optical responses^1^. By engineering these individual structures, or “meta-atoms”, and properly arranging them on a surface, a wide range of desired linear optical functionalities can be achieved^2–5^.

In practice, device functionality is limited by our ability to completely control these four properties arbitrarily and independently. This limitation comes down to the challenge of engineering the individual meta-atoms with widely varying desired responses at desired frequencies within a single achievable fabrication scheme. For this reason, most of the effort in the field of metasurfaces has focused on a single property at a time. Since phase is arguably the single most important property for wavefront control, metasurfaces engineering the phase profile of a wavefront dominate the published works^1–5^. While metallic scatterers are often used due to their strong light-matter interactions^6–10^, to overcome the inherent optical losses involved with metals, lossless dielectric material platforms are commonly employed for high-efficiency phase control^11–19^.

Expanding the gamut of achievable flat optical devices requires control of more than just the phase. For this reason, recent efforts have pushed for simultaneous control of more than one parameter at a time. A number of works have shown the flexibility of controlling the phase and polarization independently, enabling devices such as polarimeters^20^, polarization-dependent lensing^13,21,22^, and polarization-dependent holography^13,15,23,24^. Of considerable recent interest is controlling the phase at different frequencies independently, enabling multiwavelength or achromatic metasurfaces^25–29^, dispersion-engineered devices^26^, and multicolor holograms^14,30–33^.

The most general linear optical device is the hologram, originally conceived as a microscopic principle encoding the amplitude and phase simultaneously^34,35^. Due to constraints in the ability to control an optical wavefront, metasurface holography is conventionally performed with a meta-atom library that controls only the phase^36^. Recent efforts have demonstrated meta-atom geometries allowing simultaneous amplitude and phase control and explored the benefits thereof for holography^37–40^. However, these efforts have been limited in efficiency or achieve results with unnecessary complexity.

Here, we present a metasurface platform with arbitrary and simultaneous control of the amplitude and phase at telecommunication frequencies in a transmission-type device. The amplitude is controlled by varying the conversion efficiency of circularly polarized light of one handedness into the circular polarization of the opposite handedness via structurally birefringent meta-atoms, while the phase is controlled by the in-plane orientation of the meta-atoms. This approach is a generalization of the well-studied metasurface platform employing the “geometric” or “Pancharatnam-Berry” phase, and we stress the conceptual and practical simplicity of this approach for achieving simultaneous and independent control of the amplitude and phase. This approach is easily generalizable to visible frequencies, and the fabrication of these dielectric metasurfaces is CMOS compatible. To demonstrate the advantage of simultaneous amplitude and phase control, we compare computer-generated holograms implemented with phase-and-amplitude (PA) metasurfaces and holograms implemented with phase-only (PO) metasurfaces and show that only the former are capable of creating artifact-free holographic images. To demonstrate the ability of PA holography to enable artistically interesting and complex scenes, we create metasurface holograms to generate high-fidelity three-dimensional (3D) holographic objects with distinct surface textures. To explore the utility of having two degrees of freedom per pixel, we create metasurfaces controlling both the amplitude and phase at the object plane and create a metasurface that has a grayscale image in the amplitude distribution and whose phase distribution produces a distinct holographic image at the object plane. Finally, we extend this simple scheme to include structural dispersion engineering of meta-atoms and demonstrate control of the phase and amplitude at two colors simultaneously.

## Results

A long-employed approach for spatially varying the phase of light is to use the geometric phase^16,18,41^, which is associated with the orientation of the linear polarization basis used to decompose circularly polarized light and can be simply altered by changing the orientation of the “fast axis” of a birefringent material. In the context of metasurfaces, “structural birefringence” is realized with metallic or dielectric scatterers with a different optical response in one in-plane direction compared to the orthogonal in-plane direction, and the orientation of these in-plane directions is tuned to control the phase of output circularly polarized light.

The operation of this metasurface on a wavefront is best described by using the Jones calculus^42^. In metasurfaces based on the geometric phase, the outgoing polarization state is modified from an incoming state as:

1 $$\left| {\psi _2} \right\rangle = {\mathrm{\Gamma }}\left( { - \alpha } \right)M{\mathrm{\Gamma }}\left( \alpha \right)\left| {\psi _1} \right\rangle$$

where $\left| {\psi _1} \right\rangle$ and $\left| {\psi _2} \right\rangle$ are Jones vectors in an (*x,y*) basis describing the incoming and outgoing polarization states, respectively, ${\mathrm{\Gamma }}\left( \alpha \right)$ is the 2 × 2 matrix rotating a unit vector in-plane by an angle *α*, and *M* is a matrix accounting for the outgoing amplitudes (*A*_*0*_ and *A*_*e*_) and phases (*ϕ*_*0*_ and *ϕ*_*e*_) for light polarized along the ordinary and extraordinary axes, respectively:

2 $$M = \left[ {\begin{array}{*{20}{c}} {A_oe^{i\phi _o}} & 0 \\ 0 & {A_ee^{i\phi _e}} \end{array}} \right]$$

Here, we consider the accumulated phase to be due to propagation within a meta-atom, which can be thought of as a short, vertically oriented dielectric waveguide, and assume unity transmittance (or forward scattering efficiency, *η*_*forward*_) for both polarizations, which corresponds to *A*_*0*_ = *A*_*e*_ = 1. We can simplify *M* and write the relevant phases in terms of the effective refractive indices *n*_*0*_ and *n*_*e*_, meta-atom height *d*, and free-space wavevector *k*_*0*_ = 2*π*/*λ* corresponding to wavelength *λ*:

3 $$\phi _{o,e} = k_0n_{o,e}d$$

**Fig. 1:**
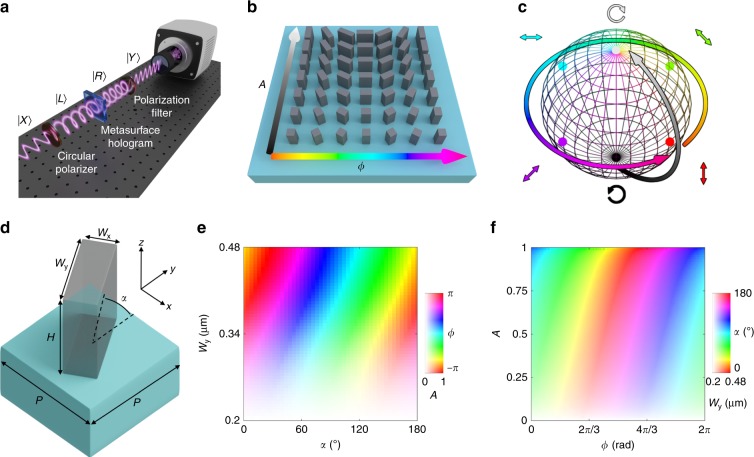
Two degrees of freedom enable independent and complete control of the optical amplitude and phase. **a** Schematic of the holographic experiment: circularly polarized light is partially converted by the metasurface to its opposite handedness and is then filtered by an analyzing polarization filter before forming an image on the camera. **b** Geometrical parameters of the meta-atoms sweep the amplitude (black-white gradient axis) and phase (rainbow axis) of the output signal. **c** The meta-atoms in **b** can take incident left circularly polarized light (south pole) to any other point on the Poincaré sphere with near-unity efficiency representing two independent degrees of freedom controlled by the metasurface. **d** Geometric parameters of a meta-atom. **e** Full-wave simulations varying *W*_*y*_ and *α* for *H* = 800 nm, *W*_*x*_ = 200 nm, *P* = 650 nm, and *λ* = 1.55 μm. The colormap depicts the amplitude, *A*, of converted light by the saturation and the phase, *ϕ*, by the hue. **f** “Look-up table” inverting an interpolated version of (**e**) to specify the values of *W*_*y*_ (saturation) and *α* (hue) required to achieve a desired *A* and *ϕ*

We take the incident polarization state to be circularly polarized light of one handedness (here, left circularly polarized, or LCP, with the Jones vector denoted as $\left| L \right\rangle$) and the signal (outgoing) state to be the opposite handedness (here, right circularly polarized, or RCP, with the Jones vector denoted as $\left| R \right\rangle$). As schematically depicted in Fig. 1a, a polarization filter in the experimental setup selects only the RCP component of the outgoing wave, yielding a signal, *S* (see Supporting Information Section S1 for a detailed derivation):

4 $$S = \left\langle R \right|{\mathrm{\Gamma }}\left( { - \alpha } \right)M{\mathrm{\Gamma }}\left( \alpha \right)\left| L \right\rangle = i\sin \left( {\frac{{k_0d\left( {n_o - n_e} \right)}}{2}} \right)\times \exp \left( {i\left( {\frac{{k_0d\left( {n_o + n_e} \right)}}{2} + 2\alpha } \right)} \right)$$

This signal is therefore a complex value with both an amplitude and a phase. The amplitude is solely dependent on the sine term, the argument of which depends in particular on the degree of birefringence of the meta-atom, (*n*_*0*_ − *n*_*e*_). This amplitude can also be thought of as the conversion amplitude, that is,

5 $$\eta _{{\mathrm{conversion}}} = \sin \left( {\frac{{k_0d\left( {n_o - n_e} \right)}}{2}} \right)$$

from LCP to RCP. It is unity when $\left| {n_0 - n_e} \right|d = \lambda /2$ and is zero when the meta-atom has no birefringence, that is, $\left| {n_0 - n_e} \right|d = 0$. Every other amplitude in between is achievable by varying the degree of birefringence between these two extremes.

The conventional choice for metasurfaces based on the geometric phase is to tune the birefringence to the half-wave-plate condition, yielding the maximum optical amplitude. Then, the optical phase is controlled through the rotation angle, *α*. Here, we generalize this approach by creating a meta-atom library utilizing both *α* and the degree of birefringence of the meta-atoms, as visualized in Fig. 1b. The amplitude is controlled entirely by the degree of form birefringence, while the phase is a sum of the propagation phase, $\frac{{k_0d\left( {n_o + n_e} \right)}}{2}$, and the geometric phase 2*α* (Eq. 4). In this way, both the amplitude and phase can be completely and independently controlled.

The action this meta-atom library performs on input circularly polarized light can be visualized by paths along the Poincaré sphere (Fig. 1c). The incident LCP light is placed at the south pole of the Poincaré sphere. The birefringence of the meta-atom determines the “latitude” of the output state, while the rotation angle *α* determines the “longitude” on the Poincaré sphere. In this way, incident LCP light can be converted into any polarization state (see Supporting Information Section S2). With the addition of a polarization filter (selecting for RCP light and absorbing the remaining LCP light), the output state on the Poincaré sphere is mapped to the amplitude and phase of the RCP light.

For a proof-of-concept implementation, we choose an operating wavelength of λ = 1.55 μm and a CMOS-compatible platform of amorphous silicon (*a*-Si) metasurfaces on fused silica substrates. The metasurface holograms consist of a square lattice of meta-atoms with rectangular in-plane cross-sections, with the geometric parameters defined in Fig. 1d. A lattice constant of *P* = 650 nm and meta-atom height of *d* = 800 nm are chosen so that for a large variation of *W*_*x*_ and *W*_*y*_ (in-plane widths of the meta-atoms), the forward scattering amplitudes, *η*_forward_, for both *x* and *y* polarized light are near-unity (see Supporting Information Section S3). This ensures that *A*_*0*_ ≅ *A*_*e*_ ≅ 1 and that the conversion amplitude is identical to the amplitude of the output signal:

6 $$\left| S \right| = \eta _{{\mathrm{forward}}}\eta _{{\mathrm{conversion}}} \cong \sin \left( {\frac{{k_0d\left( {n_o - n_e} \right)}}{2}} \right)$$

To find suitable combinations of *W*_*x*_ and *W*_*y*_ of the target meta-atom library, finite-difference time-domain (FDTD, Lumerical Solutions) simulations are performed, and a contour through the simulated parameter space is chosen that closely satisfies the condition of *η*_forward_ = 1 while providing *η*_conversion_ that continuously varies from 0 to 1. The specific chosen contour has *W*_*x*_ = 200 nm and *W*_*y*_ varying from 200 to 480 nm (refer to Supporting Information Section S3).

The amplitude and phase of the RCP component of the output are then recorded for each combination of *W*_*y*_ and *α*, as shown in Fig. 1e. Note that the converted amplitude is essentially independent of the orientation angle, indicating that the effect of coupling between neighboring meta-atoms on effective refractive indices *n*_0_ and *n*_e_ is negligible and validating the absence of *α* in Eq. 6. For ease of use, the simulation results are inverted into a “look-up” table (Fig. 1f) (see Supporting Information Section S4 for this process), wherein a desired amplitude and phase combination can be converted to the required geometric parameters, *W*_*y*_ and *α*. The successful inversion from Fig. 1e, f numerically demonstrates the arbitrary control of the amplitude and phase achieved by the meta-atom library.

**Fig. 2:**
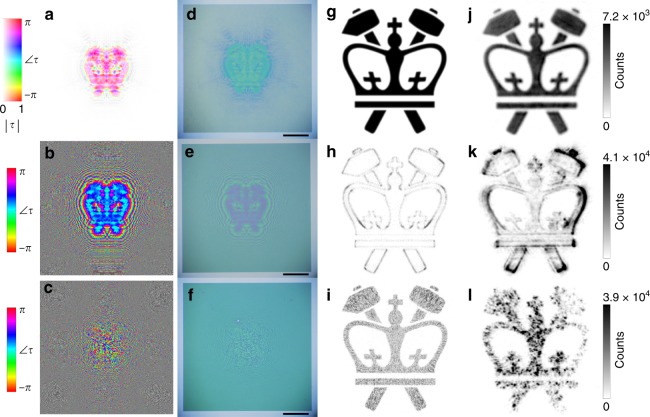
Experimental comparison of phase-amplitude (PA, top row), phase-only (PO, middle row), and Gerchberg-Saxton (GS, bottom row) holography. **a**–**c** The required amplitude and phase across each metasurface, where the saturation of the image corresponds to the amplitude and the hue corresponds to the phase. **d–f** Optical images of fabricated holograms. Scale bars are 150 µm. **g**–**i** Simulated holographic reconstructions. **j–l** Experimental holographic reconstructions, with counts shown for comparison

**Fig. 3:**
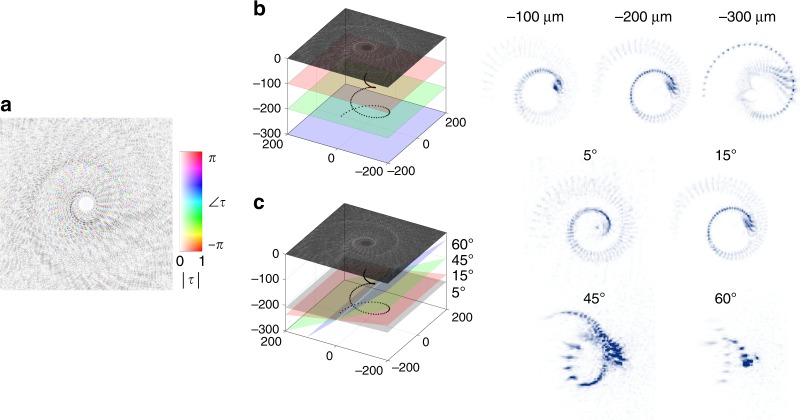
Experimental demonstration of depth and parallax in a 3D holographic object. **a** Complex transmission function, *τ*, of a 3D coil that is 400 × 400 μm in size. **b** Experimental reconstruction of the coil at three depths, showing the 3D nature of the coil. The approximate focal plane positions relative to the metasurface plane and point sources representing the coil are shown for reference. Note that the focal planes are tilted by approximately 15° to the metasurface to reduce spurious back reflections that were present. **c** Reconstruction of the coil at varying observation angles with approximate focal planes for reference, demonstrating parallax

**Fig. 4:**
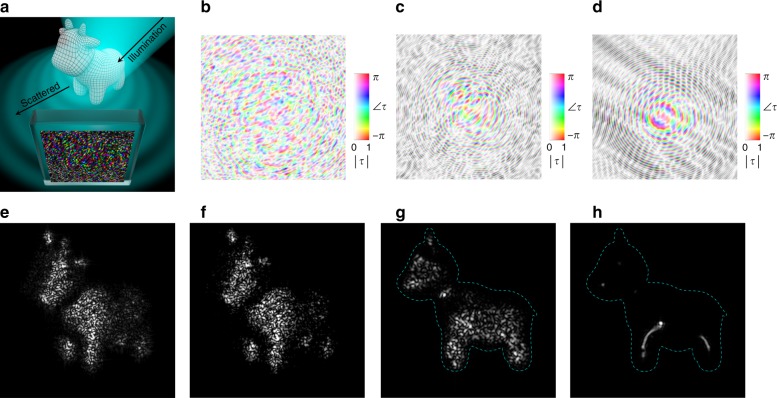
3D computer-generated holographic objects with controlled surface textures. **a** Schematic depicting the calculation of the complex transmission function, *τ*, of a metasurface hologram to generate a complex 3D holographic object (a cow). An illuminating beam is scattered by the mesh of the cow and undergoes interference at the plane of the metasurface. **b** *τ* for the cow with a rough surface texture at the viewing angle shown in **e** and **f**. **c** *τ* for the cow with a rough texture at the viewing angle shown in **g**. **d** *τ* for the cow with a smooth texture at the viewing angle shown in **h**. **e** Simulated reconstruction of the cow, showing excellent agreement with **f** the experimental reconstruction with a diode laser. **g**, **h** Simulated reconstructions from a different perspective, showing the effect of surface textures on the reconstruction; for the smooth cow in **h**, only the specular highlights are apparent

**Fig. 5:**
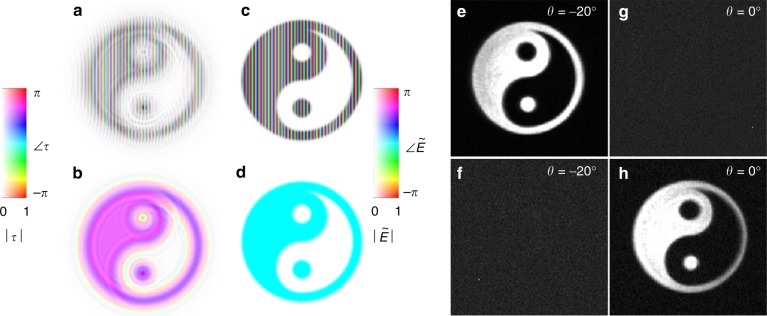
Controlling the amplitude and phase of holographic images simultaneously. **a**, **b** Complex transmission functions, *τ*, of two holograms. **c**, **d** Simulated reconstructed complex amplitudes, $\tilde E$, of **a**, **b**, yielding holographic images with identical intensity distributions but distinct phase distributions: one has a phase gradient and the other has a uniform phase. **e**, **f** Experimental holographic reconstructions corresponding to **a**, **b** at an observation angle of *θ* = −20° from the surface normal. **g**, **h** Experimental holographic reconstructions corresponding to **a**, **b** at an observation angle of *θ* = 0°. The dependence on observation angles is proof that the holographic images have distinct phase gradients, which correspond to distinct far-field projection angles

**Fig. 6:**
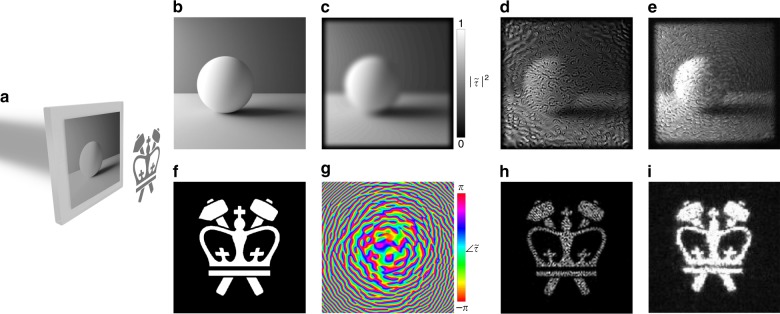
Two images encoded by a modified Gerchberg-Saxton algorithm allowing a grayscale amplitude at the metasurface plane. **a** Schematic showing the illumination of a metasurface, with an amplitude profile depicting an image of a sphere on a flat surface. The phase profile of the metasurface (not shown) encodes a holographic object (Columbia Engineering logo) at the object plane (3 mm away). **b**, **f** Target intensity profiles (before blurring) at the metasurface and object planes, respectively. **c**, **g** Intensity and phase profiles encoded on the metasurface. **d**, **h** Simulated reconstructions when focused onto the metasurface and object planes, respectively. **e**, **i** Experimental reconstructions when focused onto the metasurface and object planes, respectively. The metasurface has side lengths of 780 μm, and the logo is ~250 μm across

To showcase the complete control of the amplitude and phase, computer-generated holograms (CGHs) are implemented experimentally. Five CGHs are demonstrated: the first generates a two-dimensional (2D) holographic image and demonstrates improved fidelity of the image produced with PA holography over those produced with two versions of PO holography (Fig. 2); the second is a CGH that creates a simple 3D holographic scene consisting of a collection of points and demonstrates 3D holography by the dependence of the reconstructed holographic scene on the focal plane and observation angle of the imaging optics (Fig. 3); the third CGH demonstrates the faithful reconstruction of a complex 3D holographic object (Fig. 4); the fourth demonstrates the ability to separately encode the phase and amplitude at the object plane (Fig. 5); and the fifth demonstrates the encoding of a holographic image with the phase distribution of a grayscale hologram, itself an image in the amplitude distribution (Fig. 6). Detailed information about the CGHs can be found in Supporting Table S1.

To generate the 2D CGH, a target image (the Columbia Engineering logo) is discretized into dipole sources with amplitudes of 1 (corresponding to the area inside the logo) and 0 (corresponding to the background) and a uniform phase. A Gaussian filter is then applied to blur the sharp boundaries between the values of 0 and 1, as these boundaries represent information encoded at higher momenta than the free-space momentum (see Supporting Information Section S5 for the effect of skipping this blurring step). The interference of these dipole sources is recorded at a distance *D* = 750 μm from the target image, which corresponds to the location of the metasurface that will reconstruct this target image. The result is a complex transmission function, $\tilde \tau (x,y)$, required at the metasurface plane:

7 $$\tilde \tau \left( {x,y} \right) = \mathop {\sum }\limits_{i,j} \frac{{\exp (i\;k_0\;R_{ij}\left( {x,y} \right))}}{{R_{ij}\left( {x,y} \right)}}$$

where $R_{ij}\left( {x,y} \right)$ is the distance from the $\left( {i,j} \right)th$ dipole source to a position (*x,y*) on the metasurface. Finally, $\tilde \tau (x,y)$ is normalized: $\tilde \tau _{{\mathrm{norm}}}(x,y)$ = $\tilde \tau (x,y)$/$\left| {\tilde \tau \left( {x,y} \right)} \right|_{{\mathrm{max}}}$. For the first PO hologram, the amplitudes are simply set to unity.

For the second PO hologram, which we refer to as the GS hologram, an alternate approach (called the Gerchberg-Saxton algorithm^43^) is used, which sets amplitude responses to unity and iteratively corrects the phase at the metasurface plane to generate the desired intensity distribution of the target image. No such iteration is necessary in the PA holography, as we can faithfully reproduce both the phase and amplitude of the desired hologram, the advantages and disadvantages of which are discussed below.

The resulting $\tilde \tau (x,y)$ for the PA, PO, and GS holograms are depicted in Fig. 2a–c. The devices are fabricated using a CMOS-compatible process, described in Supporting Information Section S6. The resulting optical images of the 2D holograms are shown in Fig. 2d–f. They consist of a layer of nanostructured amorphous silicon 0.8 μm in height patterned on a fused silica substrate. The overall size of each hologram is 750 × 750 μm.

The reconstruction of each holographic image is performed both by numerical simulation (Fig. 2g–i) and experimentally (Fig. 2j–l, see Supporting Information Section S7 for experimental details). The improvement of the image quality in the PA hologram compared to either PO or GS hologram is readily apparent, reflecting the uncompromised reconstruction of a target image. The PO hologram can be seen to highlight the edges of the logo, suggesting that a role of amplitude variation in the PA hologram is to correctly modulate the amplitudes of the high spatial frequencies in the reconstructed image. This can be seen visually by comparing the $\tilde \tau (x,y)$ of PA and PO holograms: where the outer edges of the hologram for the PA (representing a large bending angle) have low amplitude, the PO hologram must have unity amplitude. The GS hologram solves this limitation of the PO hologram by employing the iterative algorithm described above. However, it appears “grainy” or “splotchy” due to unwanted destructive interference within the logo boundaries, a well-known limitation of GS holography. The dependence on wavelength for a 2D PA and PO hologram is shown in Fig. S8, demonstrating that the broad bandwidth of the geometric-phase approach extends to PA holography.

A further showcase of the capabilities of PA holography can be seen in Figs. 3 and 4, where 3D holography is demonstrated. Figure 3a shows $\tilde \tau (x,y)$ for generating a 3D coil, calculated by discretizing the coil into an array of dipole sources and recording their interference pattern at the metasurface plane. To show the depth of the 3D coil, three focal planes are chosen for experimental reconstruction, as depicted in Fig. 3b. The individual dipole sources are discernible at the farthest focal plane of 300 μm, where the distribution of the dipoles is sparsest, while at the nearest focal plane of 100 μm, they are nearly continuous. As seen in Fig. 3c, parallax is demonstrated by changing the viewing angle of the camera (maintaining normally incident light to the metasurfaces), with a recognizable image observed at an angle as high as 60° (approximate corresponding focal planes are drawn in Fig. 3c). This verifies the true holographic nature of the experiment: the reconstruction simulates looking through a window into a virtual world populated by the 3D coil.

To demonstrate the ability of PA holography to enable more artistically interesting and complex scenes, a target 3D-modeled cow is converted into a hologram and then reconstructed. Figure 4a depicts the computation of $\tilde \tau (x,y)$ for generating the cow, computed with a simulation interfering light waves scattered off the 3D surface of the cow. This method of computer-generated holography, described in Supporting Information Section S9, includes realistic physical effects such as occlusion and surface textures. In particular, rough or smooth surface textures are simulated by choosing a random or uniform distribution of scattered phase over the surface of the cow. Three $\tilde \tau (x,y)$ are calculated in this manner and shown in Fig. 4b–d. Figure 4b depicts $\tilde \tau \left( {x,y} \right)$ for a cow with a rough surface at an oblique perspective, while Fig. 4c, d depict, respectively, $\tilde \tau \left( {x,y} \right)$ for a cow with a rough and a smooth surface from an edge-on perspective.

The optical reconstruction is performed both computationally (Fig. 4e) and experimentally (Fig. 4f). The excellent agreement, even in the details of the speckle pattern, affirms the fidelity with which the PA holography platform can capture effects such as surface roughness. See Supporting Information Section S10 for details on the simulated reconstruction. Reconstruction using an LED (linewidth ~120 nm centered around 1.55 μm) shows a reduction in the speckle contrast due to the increased bandwidth and incoherence of the source (see Supporting Information Section S11).

Figure 4g, h contains the simulated reconstructions of the rough and smooth cows, respectively, with the outline of the cow shown for reference. Notably, for the smooth cow, only the specular highlights (that is, the portions of the cow where the angle of incidence of the illumination is equal to the angle of observation) are apparent, while the rough cow shows a speckle pattern nearly filling the silhouette of the cow. We note that this speckle phenomenon is physically accurate and unintuitive only because of the rarity of coherent sources as the sole illumination source in everyday experience. The agreement with physical expectations demonstrates the control of PA holography over the surface texture of complex 3D holographic objects. Control over the surface texture is possible because of the simultaneous control of the object amplitude and phase, which is uniquely possible in PA holography.

PO holography uses only one degree of freedom (phase) at the hologram plane to control one degree of freedom (intensity) at the object plane. PA holography has no such limitations and, as seen in Fig. 5, may separately encode the amplitude and phase of a holographic image. Figure 5a, b contains the complex transmission functions of two holograms that encode the same object intensity profiles but distinct object phase profiles (as shown in Fig. 5c, d). Therefore, not only is the fidelity of the intensity profile improved in PA holography over PO holography (as seen in Fig. 2) but also an entirely parallel channel of information (phase) can be faithfully encoded simultaneously. In this case, the phase profiles chosen are simple gradients, meaning that the holographic objects are observable from distinct angles. This is experimentally verified in Fig. 5e–h, where the holographic images are formed only if the information projected by the holograms is within the range of angles collected by the imaging objective.

Another use of the two degrees of freedom present in PA holography is to control the amplitude profiles at two separate planes rather than the amplitude and phase at a single plane. To demonstrate this, we modify the GS algorithm to enforce a grayscale amplitude distribution (instead of the conventional uniform amplitude distribution) and iteratively recover the phase required to produce a target holographic image at the object plane given the chosen nonuniform amplitude distribution. In other words, as depicted in Fig. 6a, the metasurface can be encoded with a grayscale image (Fig. 6b) while simultaneously producing a holographic image (Fig. 6f). The intensity and phase profiles of the resulting metasurface are shown in Fig. 6c, g. The experimental reconstructions (Fig. 6e, i) are in good agreement with the simulated reconstructions (Fig. 6d, h), showing recognizable target images with artifacts inherent to GS holography (destructive interference due to a lack of phase control at each plane). Supporting Video S1 shows the transformation between the reconstructed images as the focal plane of the imaging setup is adjusted between the hologram and the object planes. Supporting Information Section S14 explores the trade-offs in image quality at the two planes and the qualitatively different nature of the “speckle” at the metasurface plane (born of the phase discontinuities) compared to that at the object plane (born of the rapidly changing phase profile).

Finally, we extend this simple approach to control the amplitude and phase independently at two separate wavelengths^33^. This represents control of four wavefront parameters simultaneously at each meta-atom and therefore requires more degrees of freedom in the meta-atom design than the two degrees of freedom (aspect ratio and orientation of rectangular meta-atoms) used above. We have shown previously that structural dispersion engineering of meta-atoms by widely varying their cross-sectional shapes (while retaining rotational symmetry or four-fold symmetry) can yield a library controlling the phase of a wide range of wavelengths at a time^29^. We extend this past effort to include form birefringence in the design of meta-atoms, allowing expansive control of the phase response of the ordinary and extraordinary polarizations at two wavelengths.

**Fig. 7:**
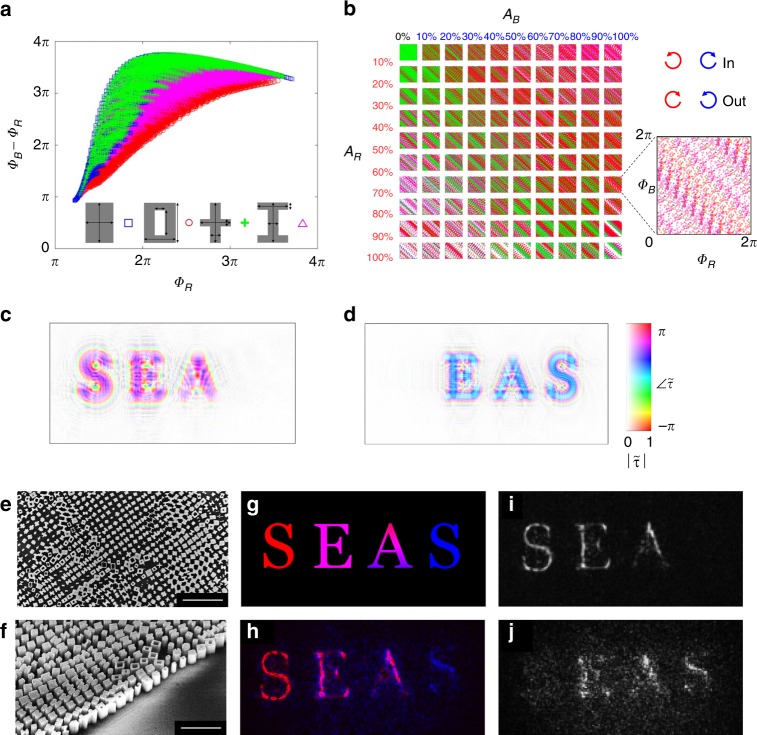
Control of the amplitude and phase at two colors simultaneously. **a** Archetypes of meta-atom cross-sections with many geometric degrees of freedom (each represented by a double-sided arrow) degenerately cover the “phase-dispersion” space of the propagation phase. **b** Visualization of the coverage of (*A*_*R*_,*A*_*B*,_*ϕ*_*R*,_*ϕ*_*B*_) by the meta-atoms in **a** with bins of 10% amplitude and circular polarization that is opposite for each color. **c** Complex transmission function of a two-color hologram for the red wavelength ($\lambda _{Red} = 1.65\;\mu m$). **d** Complex transmission function of the two-color hologram for the blue wavelength ($\lambda _{Blue} = 0.94\;\mu m$). **e** Scanning electron micrograph (SEM) of an example hologram, showing many instances of the archetypes from **a** with variable in-plane orientation angles. Scale bar is 3 μm. **f** SEM with a perspective view of the 1 μm-tall pillars in **e**. Scale bar is 2 μm. **g** Target two-color image. **h** Experimental reconstruction overlaying the separately measured pictures at the red wavelength shown in **i** and at the blue wavelength shown in **j**

Specifically, four archetypes of meta-atoms supporting form birefringence are used, each representing a subclass of meta-atoms with the geometric degrees of freedom indicated by the arrows in Fig. 7a. In addition, we (1) increase the thickness of the amorphous silicon layer from 0.8 to 1 μm to increase the range of phase dispersion resulting from propagation, (2) choose relatively widely separated wavelengths representing “red” (λ = 1.65 μm) and “blue” (λ = 0.94 μm) channels to enhance the dispersion of the optical response, and (3) set the input handedness of circularly polarized light in the “red” to be opposite that in the “blue” so that the dependence of the phase on *α* is opposite for each color (further expanding the range of responses possible).

The phase, *ϕ*_*R*_, and dispersion, *ϕ*_*B*_–*ϕ*_*R*_, due to propagation through the library of meta-atoms are depicted in Fig. 7a, demonstrating dense and degenerate coverage of this space. This degeneracy (many meta-atoms providing the same phase dispersion but different amplitudes) is key, as the amplitude must also vary widely and independently. The geometric phase is an additional degeneracy in the phase to be exploited and can be included by analytical extension of the numerical simulations. To visually explore how well the combinations of amplitude and phase (*A*_*R*_,*A*_*B*_,*ϕ*_*R*_,*ϕ*_*B*_) at the two wavelengths are achieved, Fig. 7b breaks the amplitudes into bins of (*A*_*R*_,*A*_*B*_) and plots the (*ϕ*_*R*_,*ϕ*_*B*_) within each bin. The apparent filling of every space in the (*ϕ*_*R*_,*ϕ*_*B*_) plot for every bin indicates that our meta-atom library can achieve every combination of (*A*_*R*_,*A*_*B*_,*ϕ*_*R*_,*ϕ*_*B*_) up to the precision of the bins chosen. These high-aspect-ratio meta-atoms with widely varying cross-sections therefore provide four independent degrees of wavefront control within a monolithic fabrication scheme.

For a proof-of-concept demonstration, a target two-color image (Fig. 7g) is converted as before into the required amplitude and phase on the metasurface plane at each wavelength (where the red channel of the image is used for *λ* = 1.65 μm and the blue channel of the image is used for *λ* = 0.94 μm), as depicted in Fig. 7c, d. Example scanning electron micrographs of the fabricated devices are shown in Fig. 7e, f, exemplifying the diversity of cross-sections optically encoding four independent variables at each pixel. The two-color experimental reconstruction (Fig. 7h) is acquired by aligning the results with LCP excitation at *λ* = 1.65 μm (Fig. 7i) and RCP excitation at *λ* = 0.94 μm (Fig. 7j). We note that for the “red” wavelength there is a good agreement with the target image, while the “blue” wavelength shows significant, yet poorer agreement. We attribute the difference in performance across wavelengths primarily to the poorer accuracy of the assumptions for the smaller wavelength involved in producing the meta-atom library seen in Fig. 7b. In particular, at the smaller wavelength, the structures support higher-order modes and resonances arising from the complex interactions thereof, which degrades the reliability of the “single-pass approximation”^44^. Due to the number of meta-atoms that need to be simulated (Fig. 7a represents ~60,000 meta-atoms), more accurate characterizations of the response of each meta-atom represents a daunting computational problem. We therefore restrict ourselves to the present imperfect but computationally tractable solution.

## Discussion

The advantages of PA over PO holographic metasurfaces are clear in the above demonstrations but merit a more detailed discussion. Notably, PO holography has the advantage of improved power efficiency. This comes from the fact that all of the light incident on the PO hologram contributes to the final image, unlike in PA holography, where the amplitude is continuously modulated between 0 and 1, filtering a portion of the power out. We note, however, that this reduction in efficiency is (1) highly case dependent (e.g., different illumination patterns and target holographic objects will use the input power differently) and (2) ambiguous in direct comparison to PO holography. In particular, there is a trade-off between the degree to which “ringing artifacts” can be suppressed (see Supporting Information Section S5) and the amount of power contributing to the final image: ringing artifacts (related to Gibb’s overshoot) can be reduced at the cost of lower overall efficiency (see Supporting Information Section S12). The choice of what counts as sufficient elimination of the artifacts will therefore determine the maximum efficiency of the hologram, meaning that there is no unambiguous comparison between PO and PA holography, as PO holography involves no such choice. Indeed, PO holography can be thought of as the choice within PA holography with maximal efficiency at the cost of maximal artifacts.

The cost of the increased power efficiency in PO holography is at least threefold. First, a substantially lower density of information is encoded by a PO hologram compared to that by its PA counterpart. This is because a PO hologram controls only the phase at each pixel in the metasurface plane, while a PA hologram controls both the amplitude and phase, which has the consequence that the phase at the object plane can be independently controlled by a PA hologram (Fig. 5) but not by a PO hologram. This could allow, for example, increasing the difficulty of counterfeiting in security applications by using holographic images of identical appearance (intensity) but with detectable differences in phase profile that require special equipment to decode, such as an interference-based apparatus. Furthermore, in an application involving holographic data storage, there is a multiplicative effect on the storable bits per pixel: a system capable of reading out *M* distinct values of the phase from a PO hologram would allow the storage of *M* states per pixel, while a system using a PA hologram that simultaneously reads out *N* values of the amplitude would allow the storage of *M* × *N* states per pixel.

Second, although the phase is not recorded directly by a camera or the human eye, the phase distribution on the optical wavefront contributes to the visual textures of a virtual object. As an example, a diffuse surface will have a random phase, while a glossy surface has some degree of phase uniformity. This texture detail is lost (or must be mimicked) by the PO approach but effortlessly retained in the PA approach (Fig. 4), where both the desired phase and amplitude of the holographic object are faithfully reproduced.

Third, a Gerchberg-Saxton-like algorithm is necessary to reduce the unwanted distortions to the image (seen in Fig. 2). While straightforward for reconstructing simple 2D scenes, the computational requirements make general PO holography (such as reconstructing 2D and 3D scenes^45–47^ with controlled textures) difficult and often impractical to implement, especially in dynamic holography. As shown in Figs. 3–4, no correction algorithm is necessary in 3D PA holography, which retains complete phase and amplitude information in the final 3D holographic scene. In other words, PA holography is faithful to the original imagination of holography: the PA hologram generates the wavefront produced by a virtual object and therefore is effectively a window into a virtual world.

In conclusion, we have demonstrated metasurface holograms using low-loss dielectric metasurfaces operating in transmission mode with complete and independent phase and amplitude control at one and two wavelengths. Structural dispersion engineering of meta-atoms and the geometric phase are employed to enable control of up to four wavefront parameters at each pixel of the metasurface holograms. This design principle is a simple but powerful extension of the long-employed geometric-phase metasurfaces, opening up a degree of control over optical wavefronts useful in many applications. We implemented monochromatic 2D and 3D phase-amplitude holograms using a library of meta-atoms with rectangular cross-sections supporting a wide range of form birefringence. We showed that the quality of 2D phase-amplitude holographic images was significantly improved over that of phase-only holography. We also showed that a PA metasurface may encode entirely separate profiles of the phase and amplitude at the object plane and that, for 3D holographic objects, this allows surface textures to be straightforwardly realized. We demonstrated holography using a generalized GS algorithm enabling holographic encoding with a grayscale hologram. We further implemented 2D holograms providing complete control of the optical phase and amplitude at two colors simultaneously using a library of meta-atoms with complex cross-sectional shapes. This work offers a robust and generalizable method towards realizing the primary promise of metasurfaces: to manipulate an optical wavefront at will.

## Materials and methods

The holograms are numerically generated by computing the interference of complex-amplitude point sources composing the target object at a plane to be occupied by the metasurface. As detailed in Supporting Information S9, the hologram for generating the complex 3D object is computed using Monte Carlo integration over the mesh of the cow, with the addition of a scattering phase to simulate surface textures.

As detailed in Supporting Information S10, the simulated reconstruction of holograms is performed using the convolution method in the Fourier domain using a propagation kernel of a point source and the complex transmission function of the metasurface.

Full-wave simulations of individual meta-atoms are carried out using commercial finite-difference time-domain (FDTD) software, Lumerical Solutions.

As detailed in Supporting Information S7, optical characterization is carried out by using a laser diode or light-emitting diode of the proper wavelength. The metasurface holograms are illuminated by circularly polarized light produced by a linear polarizer combined with a quarter-wave plate (Thorlabs). The light is collected by a 10 × or 100 × near-infrared objective (Mitotoyu), passed through a polarization filter (Thorlabs), and directed towards a near-infrared camera (Princeton Instruments).

Fabrication is carried out at Brookhaven National Laboratory using standard planar fabrication technologies, detailed in Supporting Information S6. Chemical vapor deposition is used to grow 800–1000 nm of amorphous silicon on a silica wafer. A double-layer of poly(methyl-methacrylate) is spun and baked at 180 °C to serve as an electron-beam resist. Electron-beam lithography (JEOL) is carried out at 100 keV and 500 pA, with a base dose of 740 µC/cm^2^ and appropriate proximity effect corrections (BEAMER). A mixture of 3:1 isopropyl alcohol to deionized water develops the exposed resist. A thin layer of alumina is deposited using electron-beam deposition, and the excess resist is stripped using a bath of N-Methyl-2-pyrrolidone (NMP) at 85 °C for 4 h. Finally, the pattern is transferred into the silicon layer by reactive ion etching.

## Supplementary information

Supporting information

Supplementary Video
