# Supplementary material for: Dielectric metasurfaces for complete and independent control of the optical amplitude and phase
Source: Light Sci Appl. 2019 Oct 9;8:92. doi: 10.1038/s41377-019-0201-7 (PMC6804926; doi:10.1038/s41377-019-0201-7)
Supplement: Supplementary file 1 — Supporting information [file 41377_2019_201_MOESM1_ESM.docx]

Supporting Information

Title Dielectric Metasurfaces for Complete and Independent Control of the Optical Amplitude and Phase

Adam C. Overvig, Sajan Shrestha, Stephanie C. Malek, Ming Lu, Aaron Stein, Changxi Zheng, and Nanfang Yu^*^

**Section S1 Derivation of amplitude and phase of RCP output from a meta-atom**

Figure S1 depicts the evolution of the Jones vector through a meta-atom for the simplified case of $\alpha=0$. To include the effects of $\alpha$, we begin with incident light, $E_{inc}= \left| \left. L \right\rangle\right.$, coming from the substrate side, with definitions of left-hand circularly polarized light ($\left| \left. L \right\rangle\right.)$ and right-hand circularly polarized light ($\left| \left. R \right\rangle\right.)$ in terms of linear polarization basis, $(\left| \left. X \right\rangle\right.,\left| \left. Y \right\rangle\right.)$:

$$\left| \left. X \right\rangle\right.=\left[ \begin{matrix} 1 \\ 0 \end{matrix} \right],$$

$$\left| \left. Y \right\rangle\right.=\left[ \begin{matrix} 0 \\ 1 \end{matrix} \right],$$

$$\left| \left. L \right\rangle\right.=\frac{1}{\sqrt{2}}\left( \left| \left. X \right\rangle\right.+i \left| \left. Y \right\rangle\right. \right),$$

$$\left| \left. R \right\rangle\right.=\frac{1}{\sqrt{2}}\left( \left| \left. X \right\rangle\right.-i \left| \left. Y \right\rangle\right. \right).$$

The state of light as a function of propagation distance $z$ through the meta-atom, $\left| \left. \Psi(z) \right\rangle\right.$ can be written as:

$$\left| \left. \Psi(z) \right\rangle\right.=\Gamma\left( -\alpha\right)M(z)\Gamma\left( \alpha\right)\left| \left. L \right\rangle\right.,$$

with

$$M(z)= \left[ \begin{matrix} {A_{o}e}^{i\phi_{o}(z)} & 0 \\ 0 & {A_{e}e}^{i\phi_{e}(z)} \end{matrix} \right],$$

$$\phi_{o}\left( z \right)=\frac{2\pi}{\lambda}n_{o}z,$$

$$\phi_{e}\left( z \right)=\frac{2\pi}{\lambda}n_{e}z,$$

and

$$\Gamma\left( \alpha\right)= \left[ \begin{matrix} cos(\alpha) & -sin(\alpha) \\ sin(\alpha) & cos(\alpha) \end{matrix} \right].$$

Taking $A_{o}=A_{e}=1$, this becomes:

$$\left| \left. \Psi(z) \right\rangle\right.=\left[ \begin{matrix} cos(\alpha) & sin(\alpha) \\ -sin(\alpha) & cos(\alpha) \end{matrix} \right]\times\left[ \begin{matrix} e^{i\phi_{o}(z)} & 0 \\ 0 & e^{i\phi_{e}(z)} \end{matrix} \right] \times\left[ \begin{matrix} cos(\alpha) & -sin(\alpha) \\ sin(\alpha) & cos(\alpha) \end{matrix} \right]\times\frac{1}{\sqrt{2}}\left[ \begin{matrix} 1 \\ i \end{matrix} \right],$$

which can be simplified to:

$\left| \left. \Psi(z) \right\rangle\right.=\frac{e^{i \frac{\phi_{o}\left( z \right)+\phi_{e}\left( z \right)}{2}}}{\sqrt{2}}\left[ \begin{matrix} \cos\left( \frac{\phi_{o}\left( z \right)-\phi_{e}\left( z \right)}{2} \right)+i\sin\left( \frac{\phi_{o}\left( z \right)-\phi_{e}\left( z \right)}{2} \right)e^{2i\alpha} \\ i \left( \cos\left( \frac{\phi_{o}\left( z \right)-\phi_{e}\left( z \right)}{2} \right)-i\sin\left( \frac{\phi_{o}\left( z \right)-\phi_{e}\left( z \right)}{2} \right)e^{2i\alpha} \right) \end{matrix} \right].$ (S1)

The action of the polarization filter is to select the RCP component of $\left| \left. \Psi(z) \right\rangle\right.$ after a propagation distance of $z=d$ (i.e., height of the meta-atom). The output from the polarization filter,$S$, is therefore calulcated by the inner product of $\left| \left. R \right\rangle\right.$ and $\left| \left. \Psi(d) \right\rangle\right.$:

$$S= \left\langle R | \Psi\left( d \right) \right\rangle= {\frac{1}{\sqrt{2}}\left[ \begin{matrix} 1 & -i \end{matrix} \right]}^{*}\times\left| \left. \Psi(z) \right\rangle\right.,$$

which simplifies to equation (4) in the main text:

$$S= i\sin\left( \frac{k_{0}d\left( n_{o}-n_{e} \right)}{2} \right)\exp\left( i\left( \frac{k_{0}d\left( n_{o}+n_{e} \right)}{2}+2\alpha\right) \right).$$

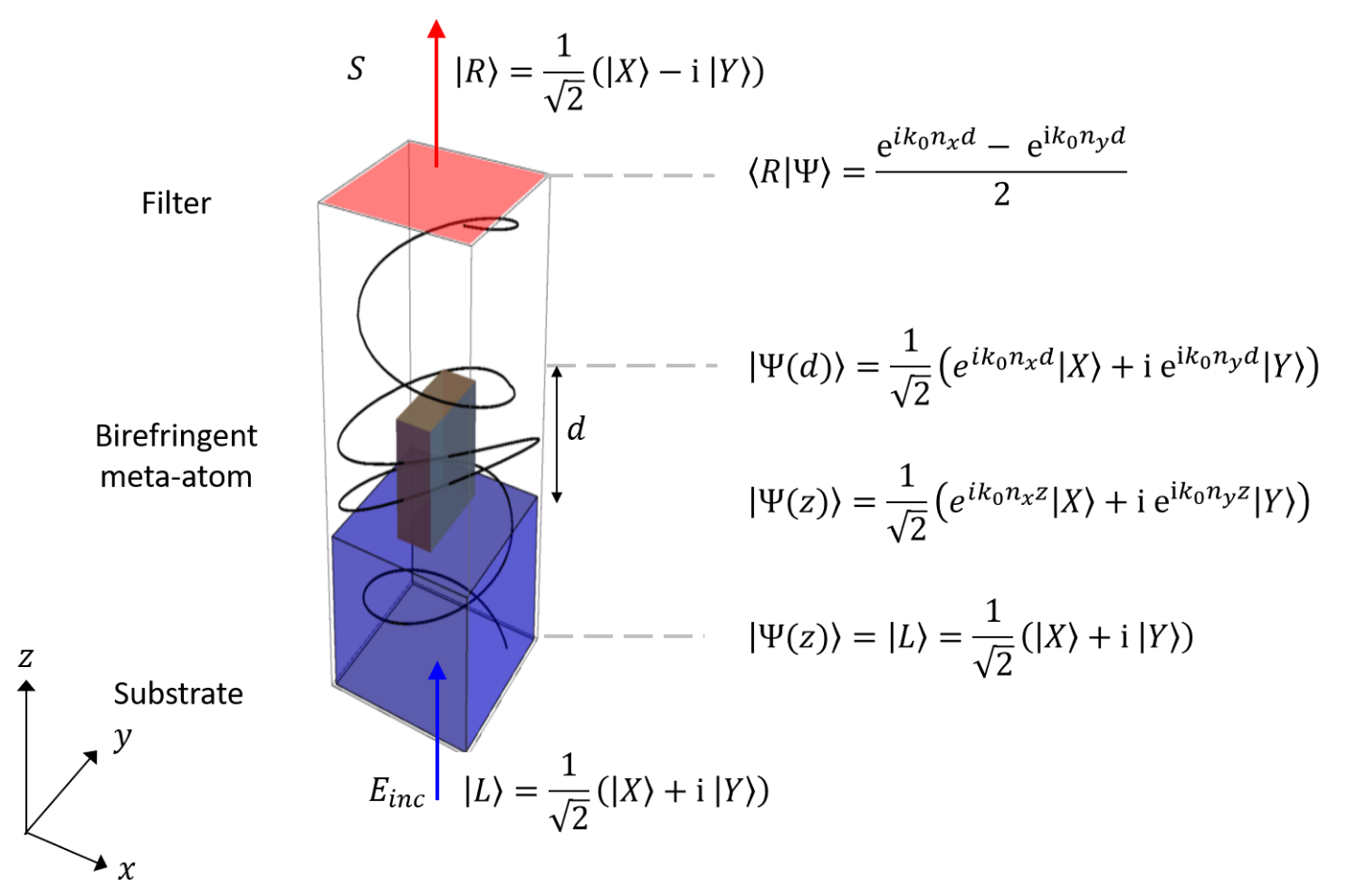


**Figure S1**. Schematic of the evolution of light through a birefringent meta-atom, with α = 0 for simplicity. LCP light is incident from the substrate side, couples into the birefringent meta-atom, evolves from LCP to a mixture of RCP and LCP (here, a complete conversion is depicted with the black curve tracing the end of the electric-field vector), and then the polarization filter selects the RCP component.

**Section S2 Meta-atom library as a polarization state converter**

We define $\eta_{conversion} =\left| S \right|= sin \left( \frac{k_{0}d\left( n_{o}-n_{e} \right)}{2} \right)$ as a measure of the birefringence of a given meta-atom. Figure S2 depicts the relationship between the output position on the Poincaré sphere and the values of $\eta_{conversion}$ and $\alpha$. The longitude, $2\psi$, and latitude, $2\chi$ of the Poincaré sphere define the two degrees of freedom determining the polarization state, and along with the intensity, $I$, are the spherical coordinates corresponding to the Stokes parameters of polarized light:

$$S_{0}=I$$

$$S_{1}=I\cos\left( 2\psi\right)\cos\left( 2\chi\right)$$

$$S_{2}=I\sin\left( 2\psi\right)\cos\left( 2\chi\right)$$

$$S_{3}=I\sin\left( 2\chi\right).$$

Complete control over the output polarization state therefore requires independent control of $\psi$ and $\chi$. As depicted in Figure S2b-d, equation S1 predicts that a meta-atom library with $\eta_{conversion}$ spanning from 0 to 1, along with $\alpha$ ranging from 0 to 180°, will be able to take incident circularly polarized light (here, LCP) into any output polarization state with unity power efficiency. Full-wave simulations (seen in Figure S2e-f and detailed in Section S3) confirm this, with Figure S2e demonstrating that the efficiency can be maintained above 96% for all meta-atoms. In both cases, it is evident that independent control of $\psi$ and $\chi$ are achieved through $\alpha$ and $\eta_{conversion}$, respectively.


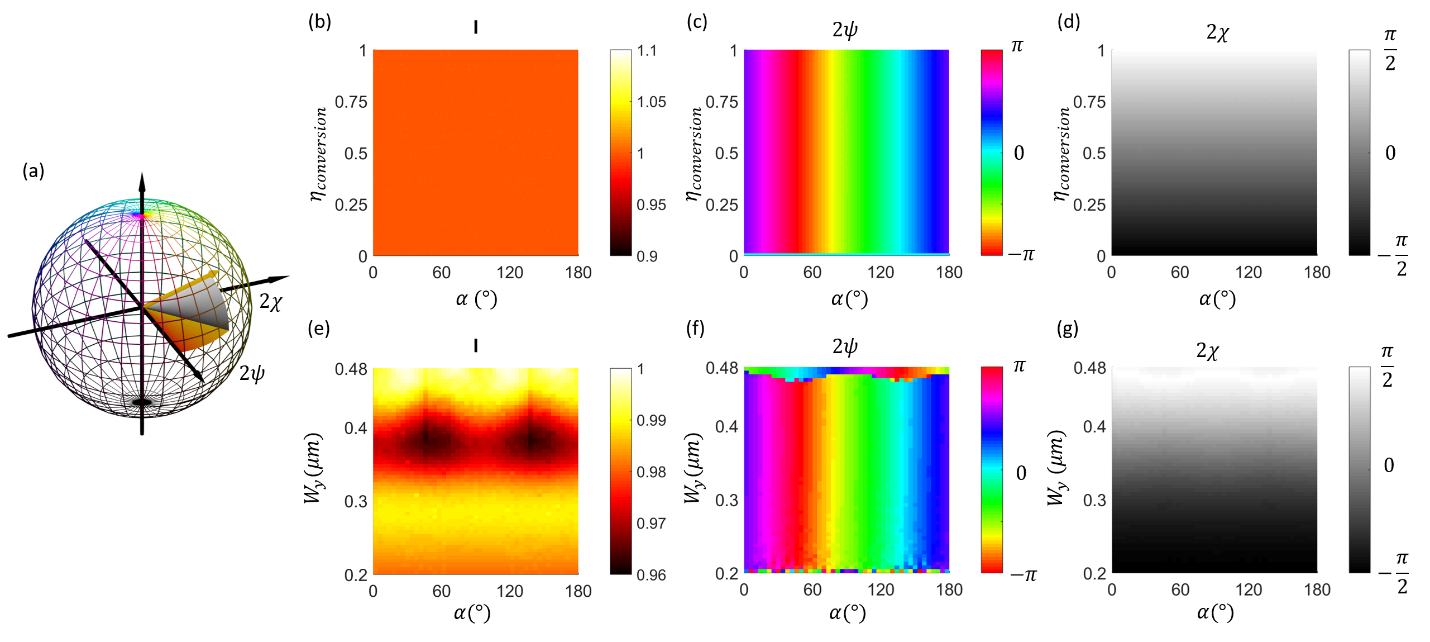


**Figure S2**. Achieving any output polarization state, visualized by the Poincaré sphere. (a) Poincaré sphere, with definitions of longitude, $2\psi$, and latitude, $2\chi$. Map of the Intensity, I (b), longitude (c), and latitude (d) predicted by equation S1, as a function of rotation angle, $\alpha$and conversion amplitude, $\eta_{conversion} = sin \left( \frac{k_{0}d\left( n_{o}-n_{e} \right)}{2} \right)$. Map of the simulated Intensity (e), longitude (f), and latitude (g) achievable by the meta-atom library. The meta-atoms are made of amorphous silicon on fused silica substrates; the lattice constant is $P=650 nm$, the meta-atom height is $d =800 nm$, and one edge of the rectangular cross-section of the meta-atom is $W_{x}=200nm$, while the other edge $W_{y}$ varies from $200 nm$ to $480 nm$; see Figure S3a for the definition of these geometric parameters. Note the complete and independent control that $\alpha$ and $W_{y}$ provide on longitude and latitude, respectively. Also note that the discontinuity in (f) is due to overshooting the north pole, with a difference of $\pi$ to the value of longitude.

**Section S3 Full-wave simulations of meta-atom library**

While the physical picture described in Section S1 predicts full amplitude and phase control, the precise geometric parameters capable and practical to achieve such control must be found by numerical methods. Toward this end, full-wave simulations (FDTD, Lumerical Solutions) are carried out on the individual meta-atoms, which do not require the approximations made in the description in Section S1 (most notably, that $A_{o}=A_{e}=1$).

Figure S3a depicts the in-plane geometrical parameters to be explored numerically. The height of the meta-atoms, $d$, and the period of the lattice, $P$, are chosen to be subwavelength, but allowed to vary within that constraint. Then, with the in-plane orientation angle, $\alpha$, kept constant, the widths in the $x$ and $y$ directions, $W_{x}$ and $W_{y}$, respectively, are varied in a parameter sweep, recording the scattering (Figure S3b) and conversion efficiencies (Figure S3c). After some initial exploration, the values $d=800 nm$ and $P=650 nm$ are chosen because they not only satisfy the subwavelength condition but also yield large scattering efficiencies for a wide range of $W_{x}$ and $W_{y}$, as seen in Figure S3b. Then, a contour through this parameter space is chosen such that the conversion amplitude varies continuouslty from 0 to 1 while the scattering efficiency remains near unity (Figure S3d). Many contours could have been chosen, but for simplicity a contour with a constant value of $W_{x}=200 nm$ was chosen, allowing the contour to be characterized by $W_{y}$ alone.

Finally, to quantify the degree to which varying $\alpha$ changes the conversion amplitude, full-wave simulations are performed varying $\alpha$ for each value of $W_{y}$. The amplitude and phase of the converted light is then recorded in Figure S3e and Figure S3f, respectively. The inversion of these simulations (detailed in Seciton S4) produces a look-up table giving the required $W_{y}$ (Figure S3g) and $\alpha$ (Figure S3h) for a desired combination of amplitude and phase.


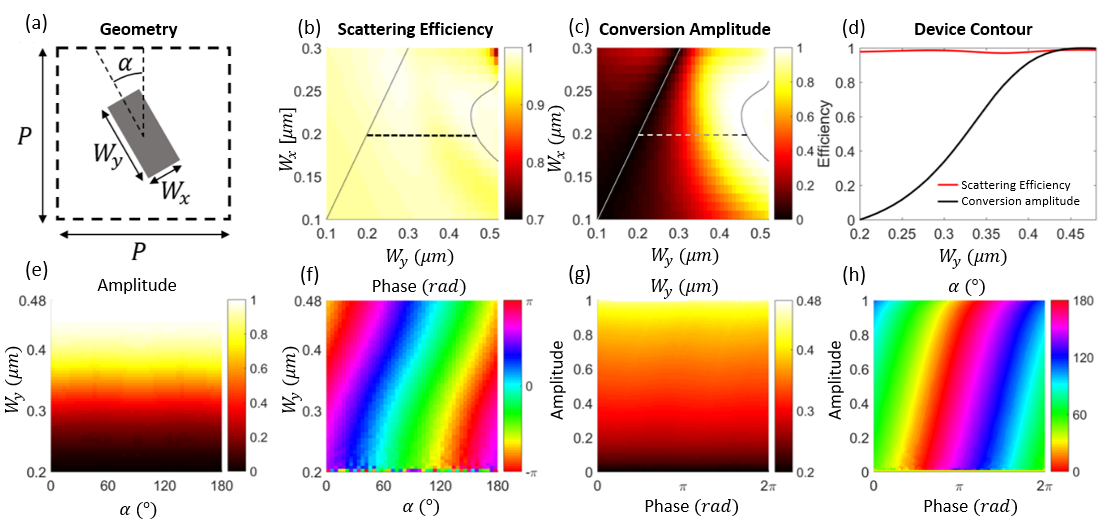


**Figure S3**. Full-wave simulations showing optical performance of the library of meta-atoms. (a) Top-view of a meta-atom showing its geometrical parameters. With $\lambda=1.55 \mu m$, $P=650 nm$,$\alpha=0$ and the meta-atom height, $d=800 nm$, a range of possible values of $W_{x}$ and $W_{y}$ are swept and the forward scattering efficiency (or transmittance) (b) and conversion amplitude (from LCP to RCP) (c) are recorded. Periodic boundary conditions are assumed in the simulations. A contour representing varying $W_{y}$ and fixed $W_{x}=200 nm$ (dashed lines in (b) and (c)) is selected to cover the full range of conversion from LCP to RCP while maintaining high scattering efficiency (>96%) (d). With LCP incident light, $W_{y}$ is swept for each choice of $\alpha$ in the range of $[0^{\circ},180^{\circ}]$, and the amplitude (e) and phase (f) of output RCP light are recorded. The results of (e,f) are inverted into “look-up” tables where for a given desired combination of amplitude and phase, the required $W_{y}$ (g) and $\alpha$ (h) can be found. The completeness of the look-up tables demonstrates the complete and independent control over the two wavefront parameters simultaneously.

**Section S4 Look-up table construction**

The process of constructing the look-up table is as follows: First, the meta-atom library simulations (Figure S3e,f) are interpolated in order to provide a library that is more continuous. This is done in lieu of additional full-wave simulations to save time, and is justified by the monotonic behavior shown in the discrete set of simulations performed. Second, a table of each combination of target phases,$\phi$, in the range of [$0, 360^{\circ}$) and amplitudes,$A$, in the range of [$0,1]$ is generated. The entries in this table take the form of a phasor: $Ae^{i\phi}$. Third, for each entry in the table, the target phasor ($A_{t}e^{i\phi_{t}})$ is compared to the achievable phasors in the interpolated meta-atom library. The geometrical parameters for the choice with minimal error is recorded along with the corresponding error ($error= \left| Ae^{i\phi}- A_{t}e^{i\phi_{t}} \right|)$. The results are shown in Figure S4. Figure S4a,b depict the look-up table constructed and Figure S4c depicts the corresponding error for each entry. The maximum error is roughly 0.011 (or 1.1%).


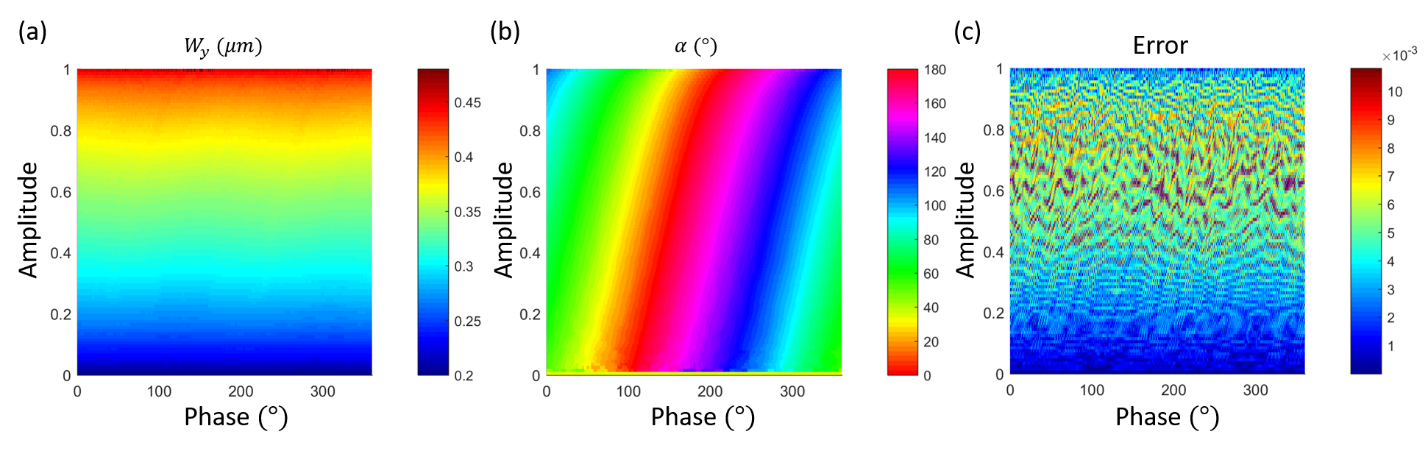


**Figure S4**. Look-up table construction. Constructed optimal choice of $W_{y}$ (a) and $\alpha$ (b) for each desired amplitude and phase combination. The absolute value of the difference in the target phasor and the closest achievable phasor is recorded for each target phasor in (c), showing a maximum error of 0.011, or 1.1%.

**Section S5 Effect of blur and numerical aperture on PA reconstruction**

Because free-space momentum of light is a fixed value constrained by the wavelength of light, there is an upper limit to the spatial frequencies encodable by a metasurface hologram. A useful quantification of this limit is the numerical aperture, $NA=sin(\theta)$, where $\theta$ is a representative range of angles across which information is encoded in the hologram. As a simple case, one can consider a metasurface lens with focal spot, $f$, and diameter, $D$, as a hologram of a single point. Then, $NA=\frac{D/2}{\sqrt{f^{2}+\left( D/2 \right)^{2}}}$ as usual. We can use this definition for 2D holograms where $f$ is the distance from the object plane to the metasurface plane and the diameter is replaced by the width, $W$, of the metasurface. For 3D holograms, we can take $f$ to be the shortest distance from the holographic object to the plane of the metasurface (for instance, the tip of the coil seen in Figure 3c of the main text). Generally speaking, the higher the $NA$, the smaller the features that can be resolved upon reconstruction for a given operating wavelength $\lambda$. The relevant parameters for the fabricated holograms are presented in Table S1.

**Table S1 Parameters of Fabricated Holograms**

| Hologram | $W (\mu m)$ | $f (\mu m)$ | $NA$ |
| --- | --- | --- | --- |
| Logo (Figure 2) | $750$ | $750$ | $0.45$ |
| Coil (Figure 3) | $400$ | $100$ | $0.89$ |
| Cow (Figure 4) | 700 | $\sim1000$ | ~0.33 |
| Yin-Yang (Figure 5) | 450 | $500$ | 0.41 |
| Sphere/Logo (Figure 6) | 780 | $3000$ | 0.13 |
| SEAS (Figure 7) | $400\times200$ | $100$ | 0.89 |

Simple concepts from Fourier analysis predict that perfectly sharp boundaries in a 2D holographic image cannot be produced by a hologram with finite $NA$ because this boundary is encoded by arbitrarily large spatial frequencies. Attempting to reconstruct a perfectly sharp boundary with a finite range of spatial frequencies results in the well known phenomenon called Gibb’s overshoot, in which amplitude ripples are apparent near the sharp boundary. To avoid such ripples, because of aesthetic considerations for instance, perfectly sharp boundaries should therefore be smoothed out to a degree such that the $NA$ and $\lambda$ of the experiment can faithfully encode the entire range of sptial frequencies represnted by the holographic object.

For our implementation, we apply a Gaussian blur to a target image (such as the Columbia Engineering Logo in Figure 2 of the main text, or the Yin-Yang symbol in Figure S5) to elimiate the presence of Gibb’s overshoot. A numerical exploration of the visual impact of a Gaussian blur with characteristic size of $b$ pixels (implemented by the Matlab function imgaussfilt($Image$,$b$) and with the physical size of a pixel being the same as the lattice spacing $P$ of the hologram) is seen in Figure S5. The metasurface is $W=400 \mu m$ in width and the object is placed at varying planes a distance $f$ away. The operating wavelength is $\lambda=1.55 \mu m$ As described above, the $NA$ is then calculated according to $NA=\frac{W/2}{\sqrt{f^{2}+\left( W/2 \right)^{2}}}$.

It is apparent from Figure S5 that for for higher $NA$, less blur (smaller *b*) is needed to remove the overshoot, consistent with the fact that a higher $NA$ metasurface encodes a wider range of spatial frequencies. However, due to the sampling theorem, a metasurface with a finite lattice spacing faces an upper limit of the value of $NA$ achieveable (beyond which a metasurface behaves like a conventional grating), resulting in a degradation in image quality regardless of the degree of blur (bottom row of Figure S5). Alternatively, a larger ratio $W/\lambda$ can be used to achieve the same image improvement without increasing the $NA$. Considering practical constraints of nanofabrication, we use metasurface dimensions less than $W=1 mm$, and correspondingly use the process depicted in Figure S5 to guide the choice of $NA$ (reported in Table S1) to produce aesthetically pleasing results for the Columbia Engineering Logo seen in Figure 2 of the main text.


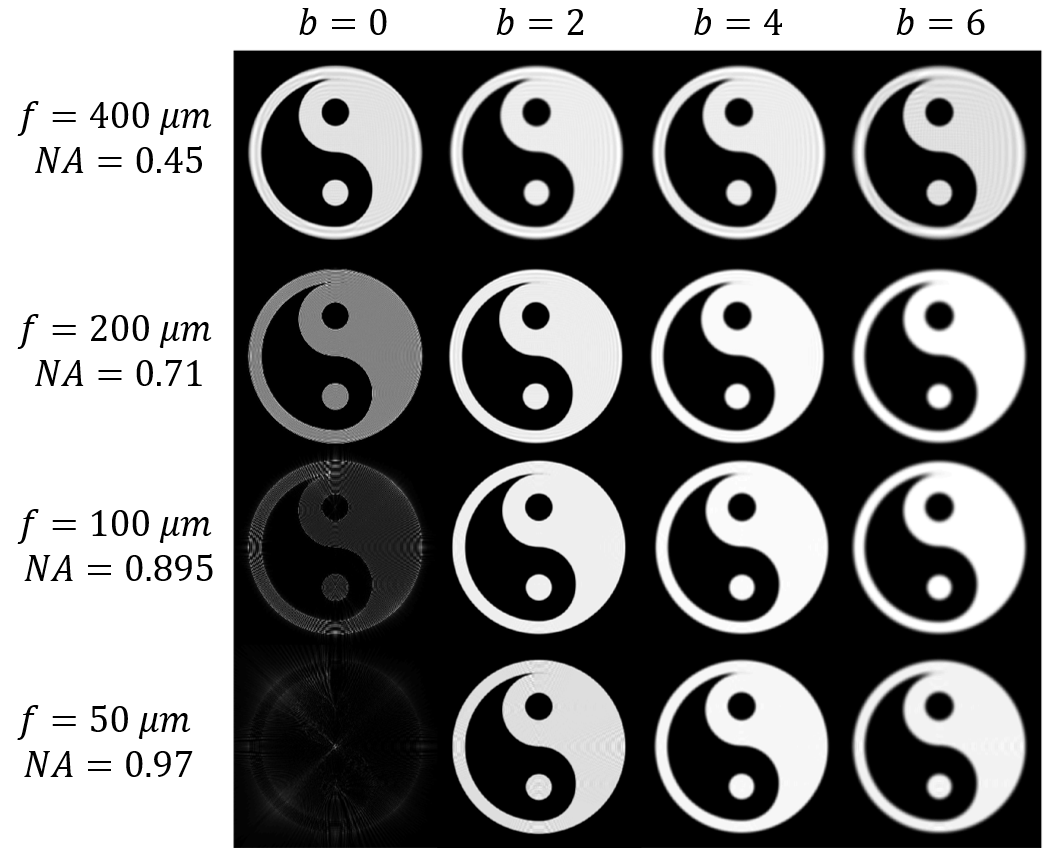


**Figure S5**. Numerical reconstruction at various combinations of numerical aperture $NA$ and degree of Gaussin blur (with characteristic size of $b$ pixels$)$. Gibb’s overshoot reduces as $b$ increases, and the magnitude of $b$ required to eliminate overshoot reduces as $NA$ increases. However, past a certain value of $NA$, the image quality degrades due to the insufficient sampling of the metasurface (due its finite lattice spacing, $P$). Careful choice is therefore required of $NA$ and $b$ such that overshoot is reduced, the image isn’t too visibly blurry, and the image is not degraded due to insufficient sampling.

**Section S6 Fabrication**

The fabrication process is summarized in Figure S6. A fused silica wafer is cleaned (with successive acetone, isopropyl alcohol (IPA), and deionized water (DIW) rinses, followed by dry nitrogen gun) in preparation of amorphous silicon growth. The amorphous silicon is grown to a thickness of 800 nm by chemical vapor deposition at a temperature of 200°C. The wafer is protected by a layer of poly(methyl methacrylate) (PMMA) spun on and baked at 180°C for 5 minutes. The wafer is cleaved into smaller pieces (roughly 1 cm ×2 cm in dimension). The protective layer is removed by an identical cleaning process as above, and replaced by a double layer of PMMA. The first layer has molecular weight of 496,000 and a dilution of 4% in anisole. The second (top) layer has molecular weight of 950,000 and a dilution of 2% in anisole. Both are spun at 4000 rpm and baked at 180°C. The first layer is baked for 10 minutes, and the second for 2 minutes.

Next, the hologram patterns are written by electron beam lithography (JEOL 6300) at a beam energy of 100 keV, beam current of 500 pA, and with a base dose of 740 µC/cm^2^ and appropriate proximity effect corrections (BEAMER). The resulting patterns are developed in a solution of 3:1 IPA:DIW for 2 minutes in a cold bath set at 5°C and then rinsed for 30 seconds in DIW at room temperature to stop development. A dry nitrogen gun is used to lightly remove remaining water from the samples.

The exposed and developed samples are then placed in a physical evaporator (LESKER) to deposit roughly 15 nm of aluminum oxide by electron beam evaporation. Lift-off is performed by dissolution of the remaining resist in N-Methyl-2-pyrrolidone (NMP) at 85°C for 4 hours. The sample is then transferred to an acetone bath and sonicated for 5 seconds to aid the completion of lift-off. After a final rinse in IPA, dry nitrogen is blown to dry the samples.

Finally, the pattern is transferred from the aluminum oxide mask to the amorphous silicon by dry etching (Oxford). The sample is attached to a silicon carrier wafer by vacuum grease (to ensure good thermal contact during etching) and placed in the etching chamber. A combination of SF_6_ and O­_2_ gases, and inductively coupled plasma power and RF power are used to control the etch rate and sidewall slope. The temperature is held at -100°C for improved sidewall smoothness.

The vacuum grease is removed by careful application of acetone and IPA by a cleanroom wipe. Light drying with a nitrogen gun finishes the removal of the vacuum grease from the back of the wafer.

The aluminum dioxide mask is left on because its very small thickness and dielectric nature make the optical impact of its presence negligible. Removal could be achieved by soaking in ammonium hydroxide, preferentially dissolving it without affecting the silicon or fused silica wafer.


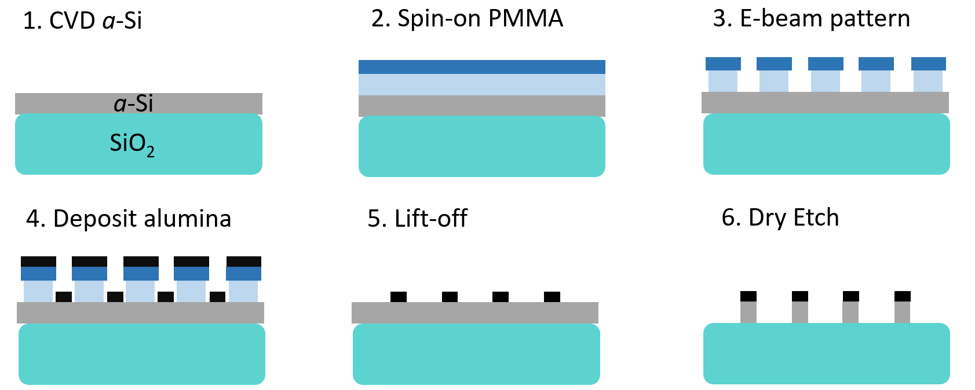


**Figure S6**. Fabrication process flow. 1. Chemical vapor deposition (CVD) of amorphous silicon (*a*-Si) on a clean fused silica wafer. 2. Spinning of double-layer PMMA electron-beam resist layer. 3. Exposure by electron-beam lithography tool and development in 3:1 IPA/DIW solution at 5°C. 4. Electron-beam deposition of alumina. 5. Chemical dissolution of remaining resist, lifting-off unwanted alumina. 6. ICP etching transferring the alumina mask pattern into the *a*-Si layer.

**Section S7 Optical characterization set-up**

Figure S7 schematically depicts the setup used for experimental reconstruction of holographic scenes by our metasurface holograms. A set of collimating optics passes circularly polarized light to the metasurface. Light is collected and analyzed by the observation optics. The observation optics and collimating optics are linked by a swivel mount allowing a varying angle, $\theta$, between the two. Due to the weight of the near-infrared (NIR) camera (Nirvana InGaAs camera, Princeton Instruments), the observation optics is stationary and the collimating optics are moved to change $\theta$. The metasurface is aligned to the axis of rotation of the swivel mount by an $(x,y,z)$ dovetail stage system attached to the collimating optics. In this way, when $\theta$ is changed, the illumination condition is fixed.

The collimating optics include a fiber collimator passing input laser light from a tunable laser source to a redirecting mirror and then to a circular polarizer before finally illuminating the metasurface from the substrate side. These collimating optics are all linked together in a cage system (cage parts are omitted for clarity in Figure S7) to the swivel mount. The metasurface is mounted on a rotation mount for control of an additional Euler angle, $\phi$.

The observation setup includes an infinity-corrected 10× objective, which collects light scattered by the metasurface, and passes it through a tube lens.Then a polarization filter and iris are used to help reduce unwanted light from reaching the camera sensor.

Note that the circular polarizer and polarization filter are identical optical elements but with opposite chirality and orientation; they are composed of a polymer polarizer cemented to a polymer quarter waveplate aligned at a ±45° angle to the fast axis of the waveplate. Light incident on the circular polarizer hits the polarizer side first, and then the resulting linearly polarized light is converted by the quarter waveplate into circularly polarized light, regardless of the polarization outputted by the fiber collimator. The “polarization filter” is the the opposite handedness of the circular polarizer, and oriented such that the quarter waveplate is illuminated first. Light of the opposite handedness than that created by the circular polarizer is therefore converted by the quarter waveplate to linearly polarized light that passes through the polarizer side, while light with the same handedness is converted by the quarter waveplate to the orthogonal linear polarization, which is absorbed by the polarizer.


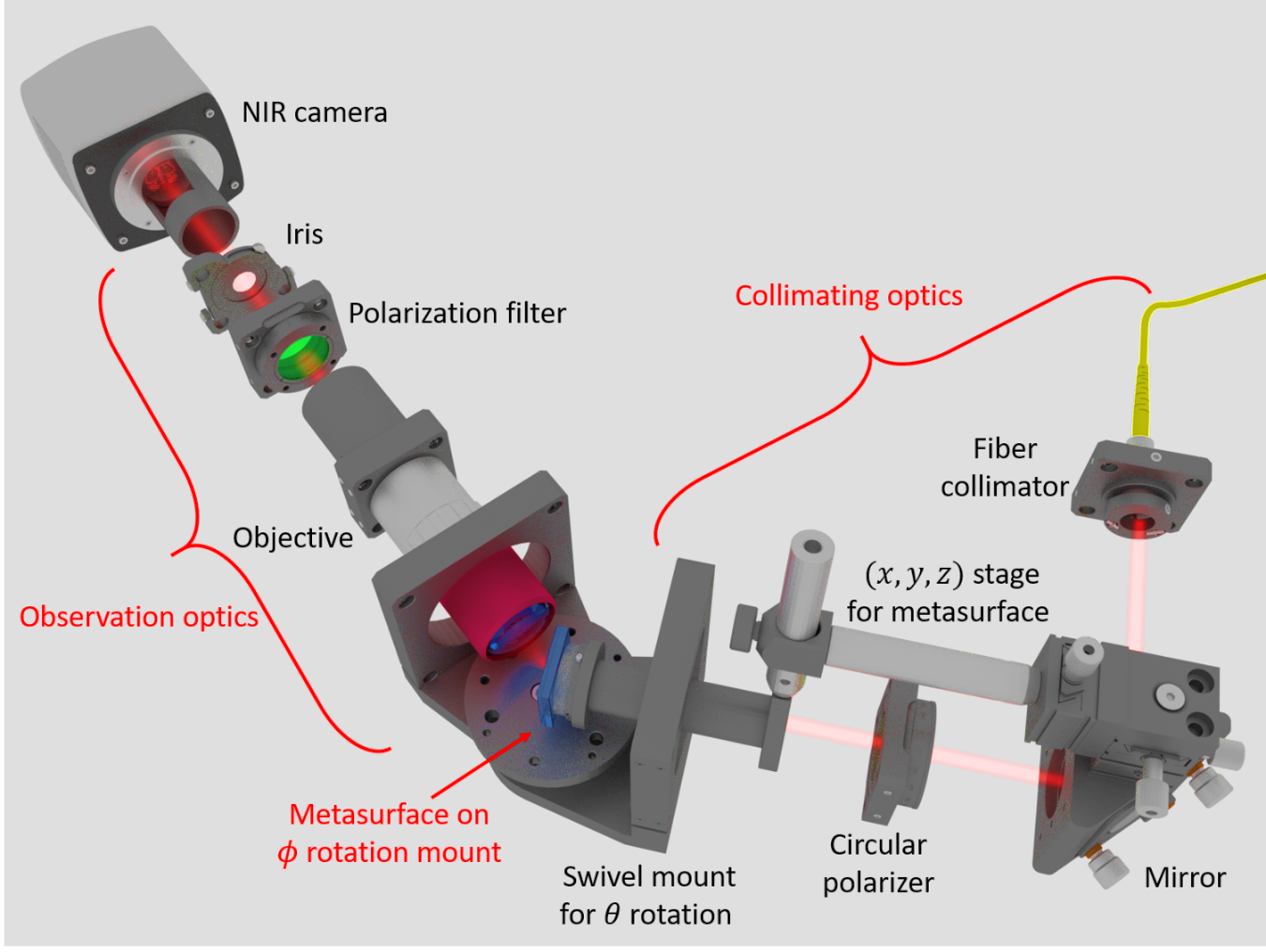


**Figure S7.** Schematic of optical setup for optical reconstruction of holographic scenes at various observation angles. Cage system parts are omitted for schematic clarity, but serve to keep the collimating condition of the light incident on the metasurface constant for varying swivel angles, $\theta$.

**Section S8 Wavelength dependence of 2D holograms**

To test the dependence on wavelength of the experimental reconstruction of 2D holographic images, light generated by a supercontinuum source (NKT Photonics) is passed through a monochromator (Horiba) and then passed to the optical setup with an optical fiber. The rest of the experiment is as depicted above. Note that the circular polarizer (ThorLabs) is designed for the operating wavelength of 1,500 nm, and has roughly 4% error in phase retardation at 1,500 nm and 1,600 nm and 8% error at 1,450 nm, which may contribute to the degradation of the holographic images slightly. A wavelength of 1,650 nm is beyond the bandwidth of the fiber used for this experiment. Notwithstanding the contributions of these errors, the bandwidth of the metasurface holograms is evidently comparable to the well-known broadband behavior of metasurfaces based on the geometric phase, as shown in Figure S8. Images are as recorded, without flipping the logo horizontally as done for the main text (to match the desired orientation).


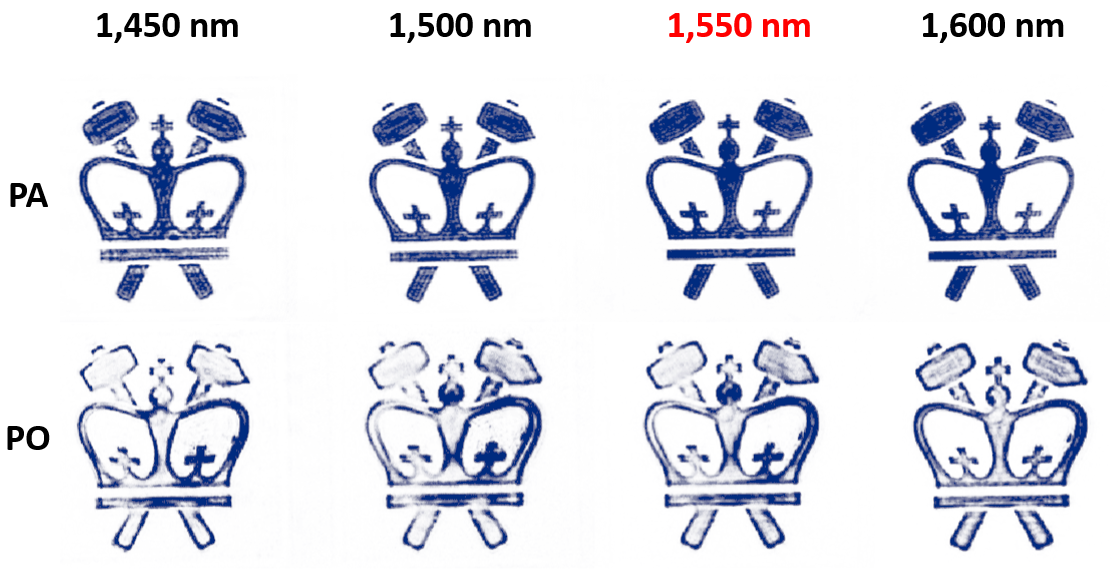


**Figure S8**. Wavelength dependence of 2D holography comparing phase and amplitude (PA, top row) to phase only (PO, bottom row) holograms for four selected wavelengths. Design wavelength of 1,550 nm is highlighted in red, and the overall bandwidth explored (150 nm) is greater than the typical of an LED centered at the operating wavelength.

**Section S9 Computer generation of the 3D hologram**

To generate the 3D hologram, we set a virtual scene wherein the cow is illuminated by an incoming plane wave. We place a hologram plane in front of the cow, and compute at every hologram pixel the optical phase and amplitude, which is a superposition of light waves reflected by the cow’s surface region that is not occluded from the incident light. We compute the phase and amplitude at each hologram pixel using Monte Carlo integration over the cow mesh: we sample points over the surface mesh, and sum the complex electric field contributed by a point source located at each sampled point. In order to account for the rough surface of the cow, we also randomly perturb the phase delay between each surface point and the pixel position. The output of this simulation process is a 2D array of complex numbers, describing the phase and amplitude distribution over the hologram.

**Section S10 Simulation of optical reconstruction**

Computer reconstruction of the 3D holographic cow mimics image formation in the eye or in a camera. We treat the CGH as an input “transparency” placed directly behind a virtual lens with a focal distance of 2.45 mm. The image plane is 9.8 mm away from this virtual lens, bringing into focus the front of the cow (which is 0.5 mm in its largest dimension), which is centered roughly 5 mm behind the metasurface. In this simulation setup, the CGH serves as a spatial light modulator that shapes the phase and amplitude of the output light field at every of its pixels. We then compute the light field intensity received on an imaging plane placed in front of the lens. The imaging plane is selected to be near the head of the cow. The simulation setup enables a fast computation of the light intensity on the imaging plane using Fourier transformation.

**Section S11 Experimental reconstruction with varying coherence**

To study the impact of coherence on the optical reconstruction, we modify the optical setup to include a light emitting diode (LED) in place of the lasers. As depicted in Figure S9, an iris is added between the LED and the metasurface (labelled MS) to allow a varied degree of spatial incoherence. When the iris is almost closed, the source is approximately spatially coherent, with a temporal coherence limited by the bandwidth of the LED (roughly Δλ=120 nm). The reconstruction in this case is comparable to reconstruction using the diode laser (Figure 3g in the main text), but slightly blurred due to the temporal incoherence. As the iris is opened, spatial incoherence adds to this blur.


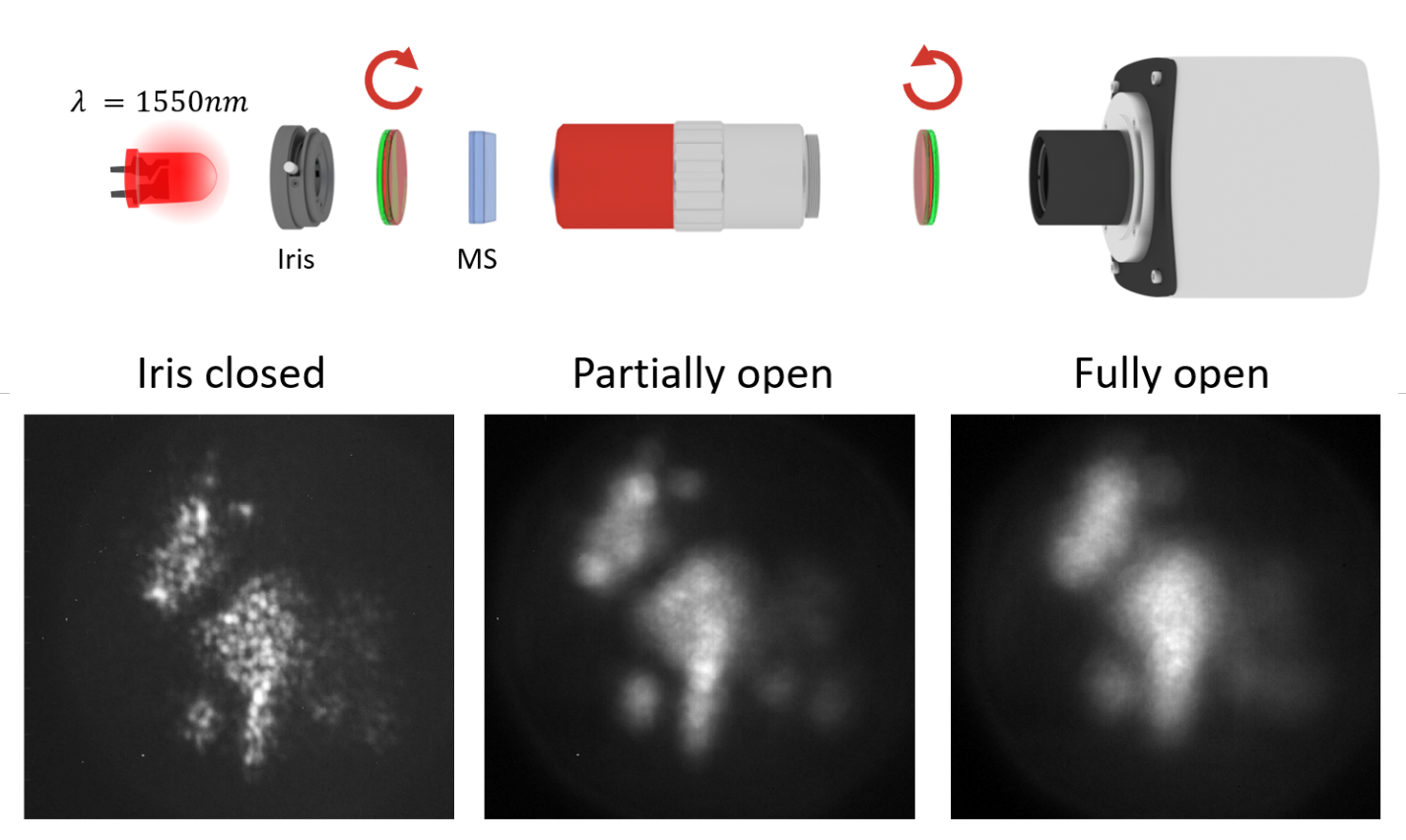


**Figure S9**. Experimental reconstruction of the cow using an LED. (Top) Schematic of the experiment using an LED and an iris for reconstruction. (Bottom) Various opening sizes of the iris yield differing degrees of spatial incoherence, resulting in a reduction in speckle as the iris is gradually opened.

**Section S12 Discussion on efficiency of holography**

It is natural to inquire as to the efficiency trade-offs between PO and PA holography, where we consider efficiency as defined by the amount of power contributing to the final image divided by the power incident on the metasurface. Of course, by design PA holography will necessarily use less of the input power than PO holography. But how much less power used is not easily generalized. It is highly case-dependent, depending on (1) the target intensity distribution of the holographic object in question, (2) the illumination pattern (e.g., shape of incident beam), and (3) the numerical aperture of the metasurface.

In particular, there is a trade-off between how much of the incident light is used and the magnitude of the ringing artifacts present upon reconstruction. Figure S10 depicts a simple case demonstrating this trade-off. A simple 1D holographic image of a blurred step function a distance $f=250 \mu m$ away from the metasurface is numerically reconstructed for metasurfaces of varying width, $W$. As $W$ increases, the amount of spatial frequencies encoded in the metasurface increases, and so the better the fidelity of the object upon reconstruction becomes (measured here by the root-mean-square (RMS) error compared to the target profile). However, it is apparent that this comes from extending an ever-decreasing tail of amplitude at the metasurface plane, meaning that normalized to the incident power (assumed here to be top-hat excitation with a width of $W$), the efficiency is dropping. Note how the efficiency monotonically decreases, while the RMS error generally trends downwards as well. A choice of RMS error must be made such that the reconstructed object will be considered of sufficiently high fidelity. This choice directly impacts the resulting efficiency, making the efficiency dependent on the quality of the holographic image, and therefore ambiguous in comparison to the case of a PO hologram.


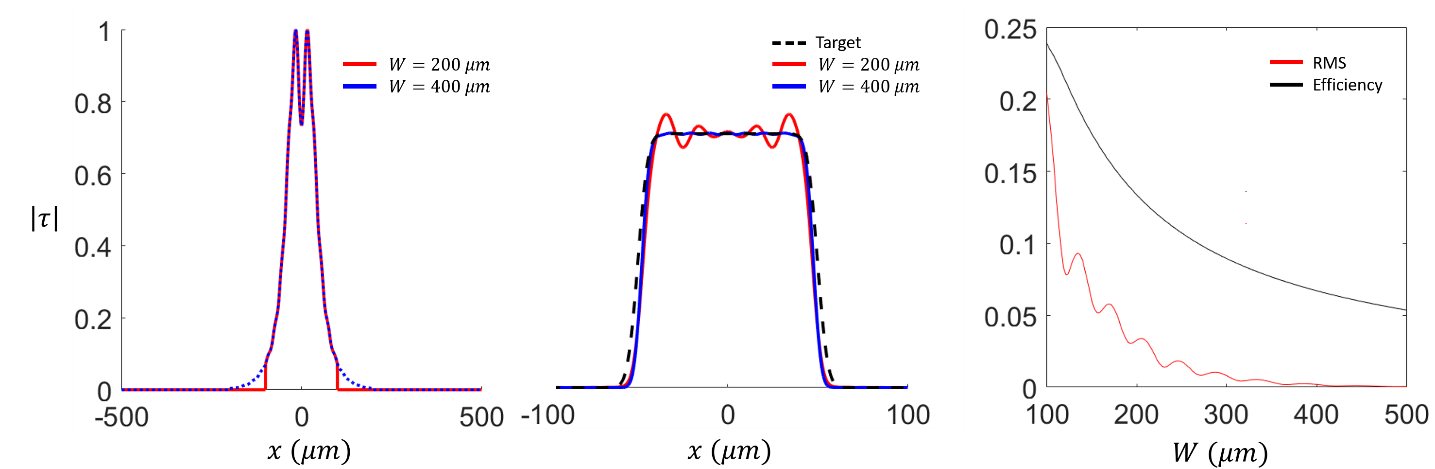


**Figure S10**. Trade-offs between Efficiency and image quality (RMS error). (left) Amplitude distribution for two example metasurfaces of different widths, $W$ (note the abrupt cutoff for the smaller metasurface). (middle) Intensity profiles of the reconstructed holographic images $250 \mu m$ away from the metasurface plane, showing worse ringing artifacts for the smaller metasurface. (right) RMS error and Efficiency as a function of $W$, showing that the amount of incident light (assumed to be top-hat excitation with lateral extent $W$) being used decreases, but the RMS error also decreases.

**Section S13 Comparison between PA, PO, and AO holography**

When only a single degree of freedom is controllable, it is generally well-known that phase is more useful than amplitude. Here, we briefly explore and compare holography using a library of meta-atoms with phase-amplitude (PA) control (with no GS algorithm) to holography using two sub-libraries, one with phase-only (PO) control and the other with amplitude-only (AO) control. In both of the latter cases, the lack of control over both phase and amplitude simultaneously requires a GS algorithm to create holograms. Figure S11 shows the results of the three cases, demonstrating that while AO control is capable of producing an image resembling the target object, the PO case is significantly improved. This confirms the presupposition that phase is more important than amplitude, and supports the interpretation in the main text that the role of amplitude is to correctly weigh the spatial frequencies of light waves produced by the metasurface: in PO, all spatial frequencies are present, and only their relative phases can be tuned; in AO, the spatial frequencie present are tuned, but their phases are all equal; in PA, the relative phases and amplitudes of all spatial frequencies are modulated.


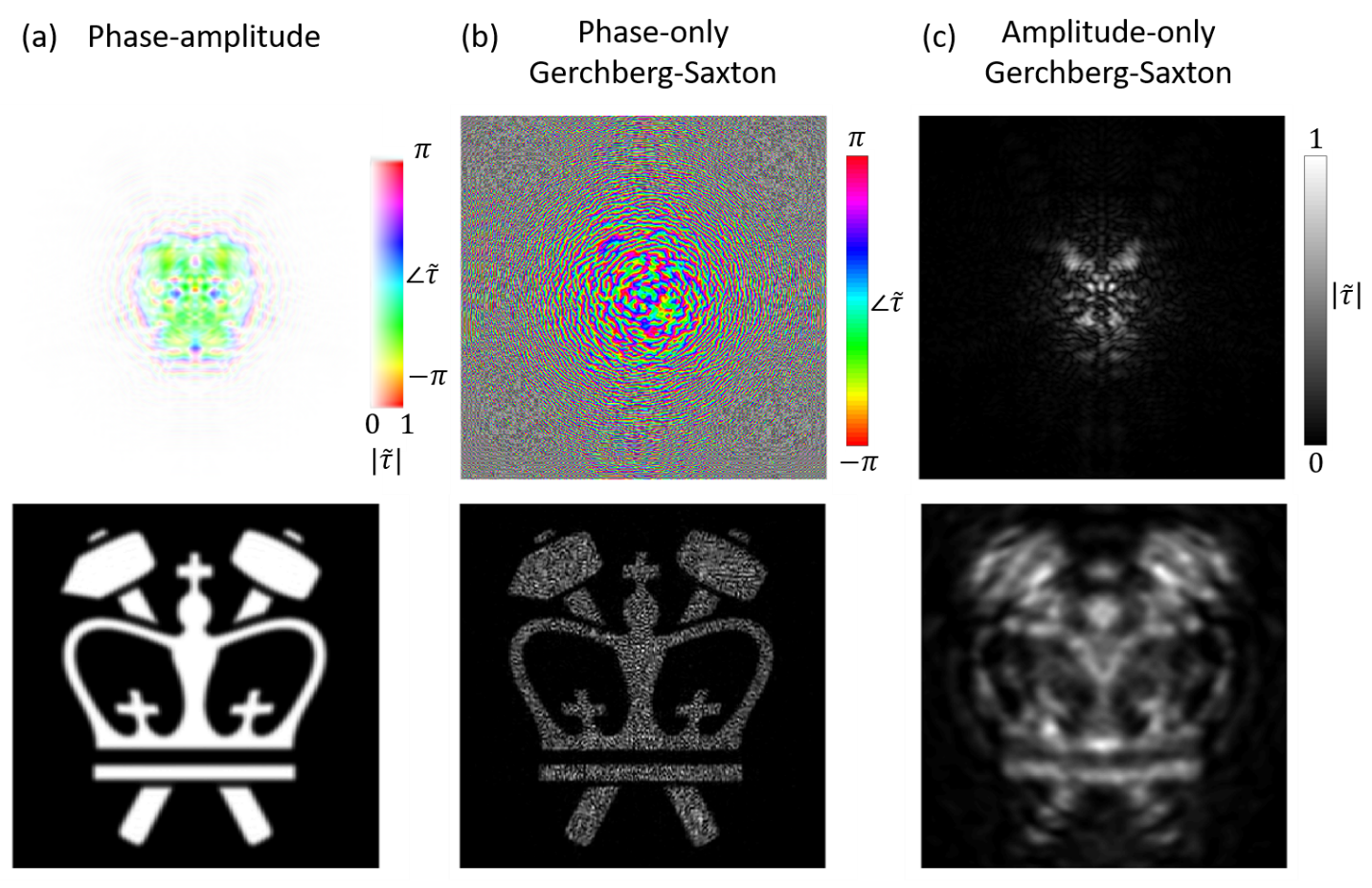


**Figure S11**. Comparison between phase-amplitude (a), phase-only Gerchberg-Saxton (b), and amplitude-only Gerchberg-Saxton (c) holography. Top row contains the metasurface complex transmission function, and the bottom row contains the simulated reconstructions. Note the degredation of the fidelity of the holographic images from left to right.

**Section S14 Gerchberg-Saxton with Phase-Amplitude control**

We briefly numerically and experimentally explore the trade-offs in the image quality at the metasurface and object planes using the GS algorithm modifed to allow a grayscale intensity mask at the metasurface plane. Figure S12 shows that as the object plane becomes closer to the metasurface plane (a distance $f$ away from the metasurface plane), the holographic image (the Columbia Engineering Logo) improves, but the image of the metasurface itself degrades. Conversely, at large $f$, the metasurface image is much improved, but the holographic image is severely degraded.

This dependence can easily be understood by considering the varying numerical aperture of the system. As $f$ decreases, the numerical aperture of the metasurface (that is, the range of spatial frequencies of the holographic object that are encoded by the metasurface) grows, meaning the object image’s quality improves. However, as $f$ reduces, the required spatial frequency of the phase variance grows. Consequently, for a small region on the metasurface plane, the phase may vary rapidly while the amplitude varies slowly, even containing phase discontinuities or sigularities. Coherently imaging such a complex field will generally yield a highly speckly image due to the destructive intereference of adjacent pixels. This destructive interference due to phase variance can be seen most clearly at large $f$, where phase varies slowly, and in only a handful of locations are there sigularities. The correspondence of such sigularities and the dark artifacts can be closely correlated by comparison across the bottom row of Figure S12. The phase sigularities generally vary across $2\pi$ around a contour circling a singualrity, while the amplitude varies slowly along the same contours. This leads to destructive interference at the (simulated) camera plane.

Two example cases from Figure S12 are implemented experimentally and shown in Figure S13. As expected, the hologram with larger $f$ has better image quality at the metasurface plane while the hologram with smaller $f$ has better image quality at the object plane. Lastly, a binary image (a Yin-Yang symbol) at the metasurface plane is shown in Figure S13c but with the same holographic object at the object plane.


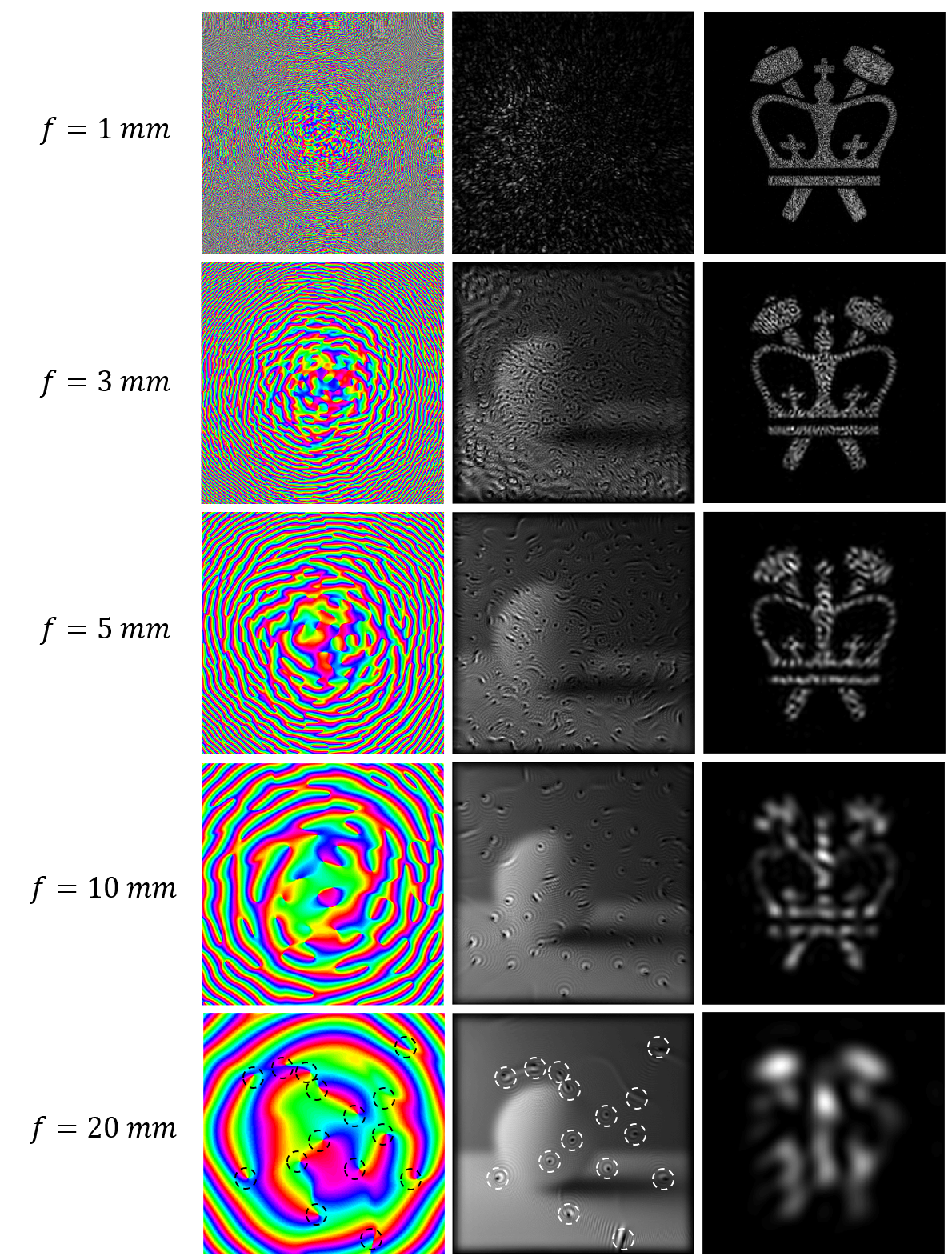


**Figure S12**. Comparison of the phase profiles (left column), simulated imaging of the metasurface plane (middle column), and simulated reconstructions of the object plane (right column) for various object plane distances, $f$, from the metasurface. Note trade-off in image quality at the two planes, increasing with $f$ for the metasurface plane and decreasing with $f$ for the object plane. Note too the correspondence between the artifacts at the metasurface plane with phase singularities in the phase profile (highlighted in the last row). This is understood by the destructive interference upon summation of pixels of approximately equal amplitudes, but varying across $2\pi$ in phase along the dashed contours shown circling the singularities.


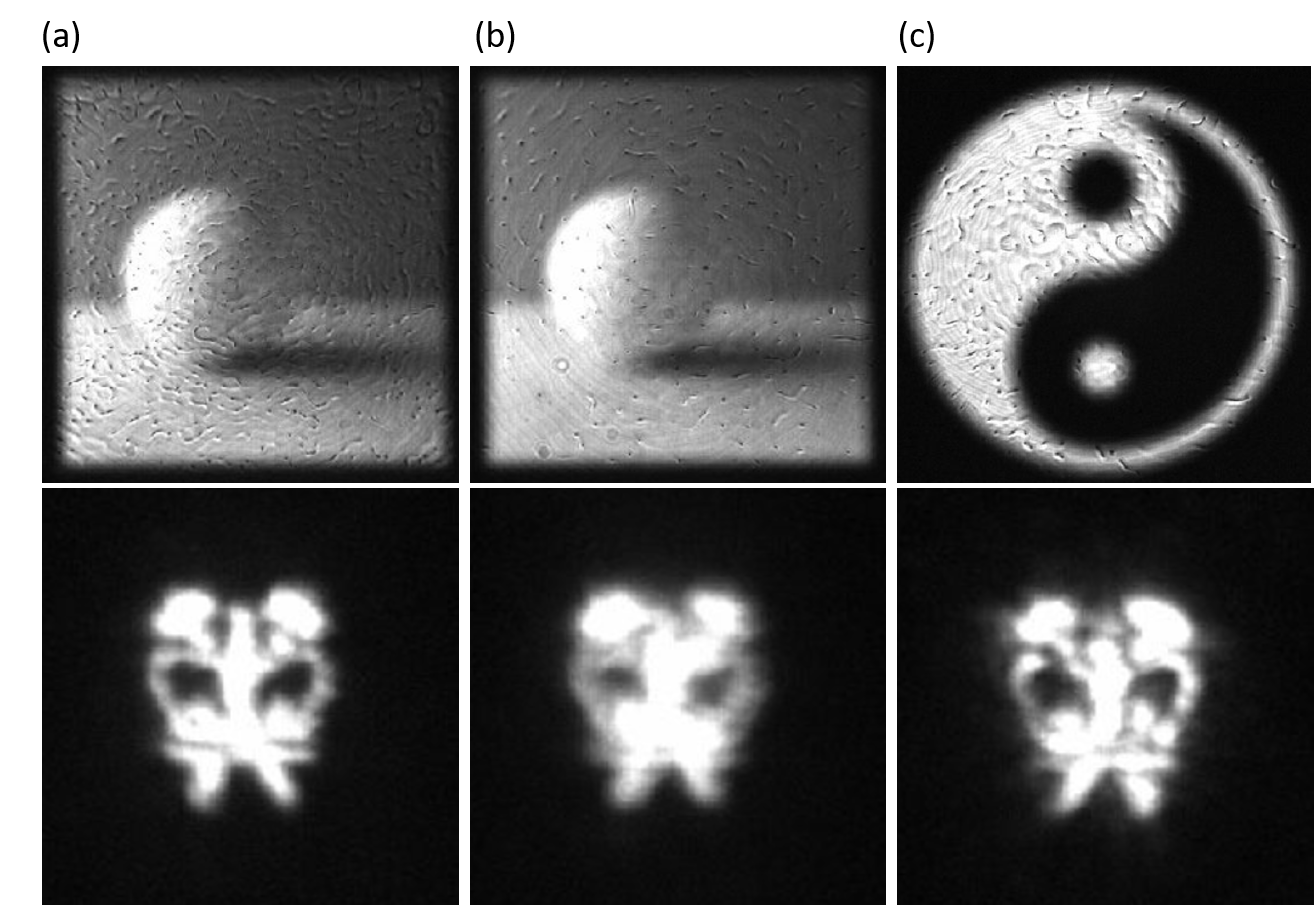


**Figure S13**. Experimental reconstructions of PA holograms using the modified GS algorithm, using LED illumination. (a) The device in Figure 6 of the main text reconstructed at the metasurface plane (top) and object plane (bottom), which is at $f=3 mm$. (b) A device similar to that in (a) but with object plane at $f=5 mm$, representing a different trade-off point from Figure S12. Note that the metasurface plane image looks improved at the expense of the object plane image. (c) Additional experimental hologram, showing a binary image at the metasurface plane (top) but the same image at the object plane (bottom), where $f=3 mm$.
